# Exploring the digesta- and mucosa-associated microbial community dynamics in the rumen and hindgut of goats from birth to adult

**DOI:** 10.3389/fmicb.2023.1190348

**Published:** 2023-06-15

**Authors:** Bibo Li, Weiqi Yin, Mingkai Lei, Xiaolong Wang, Yuxin Yang, Chunxiang Zhang, Yulin Chen

**Affiliations:** ^1^College of Animal Science, Shanxi Agricultural University, Jinzhong, China; ^2^Key Laboratory of Animal Genetics, Breeding and Reproduction of Shaanxi Province, College of Animal Science and Technology, Northwest A&F University, Xianyang, China

**Keywords:** microbiota, digesta, mucosa, gastrointestinal tract, birth to adult, goats

## Abstract

Recently, the relationship between the goat host and its gastrointestinal microbiome has emerged as a hallmark of host-microbiota symbiosis, which was indispensable for the proper physiological function that convert the plant biomass to livestock products. However, little integrative information about the establishment of gastrointestinal microflora in goats exists. Herein, we characterized the colonizing process of the bacterial community in the digesta and mucosa of the rumen, cecum, and colon of the cashmere goat from birth to adulthood to compare its spatiotemporal difference via 16S rRNA gene sequencing. A total of 1,003 genera belonging to 43 phyla were identified. Principal coordinate analysis unveiled the similarity of microbial community between or within each age group increased and gradually developed toward mature whatever in digesta or mucosa. In the rumen, the composition of the bacterial community in digesta differed significantly from in mucosa across age groups; whereas in the hindgut, there was a high similarity of bacterial composition between the in digesta and mucosa in each age group before weaning, while the bacterial community structure differed markedly between these two types of samples after weaning. Taxonomic analysis indicated that 25 and 21 core genera coexisted in digesta and mucosa of the rumen and hindgut, respectively; but their abundances differed considerably by GIT region and/or age. In digesta, as goats aged, a lower abundance of *Bacillus* was observed with higher abundances of *Prevotella 1* and *Rikenellaceae RC9* in the rumen; while in the hindgut, the genera *Escherichia-Shigella*, *Variovorax*, and *Stenotrophomonas* decreased and *Ruminococcaceae UCG-005*, *Ruminococcaceae UCG-010*, and *Alistipes* increased with age increased. In mucosa, the rumen showed microbial dynamics with increases of *Butyrivibrio 2* and *Prevotellaceae UCG-001* and decreases of unclassified_*f_Pasteurellaceae*; while the genera *Treponema 2 and Ruminococcaceae UCG-010* increased and *Escherichia-Shigella* decreased in the hindgut as goats aged. These results shed light on the colonization process of microbiota in the rumen and hindgut, which mainly include the initial, transit, and mature phases. Furthermore, there is a significant difference in the microbial composition between in digesta and mucosa, and both these exhibit a considerable spatiotemporal specificity.

## Introduction

For a long time, gastrointestinal microbes and host have coevolved to form a stable mutualistic relationship, which enables them to play an important role in the normal growth and development of animal hosts and production performance. It is given that gastrointestinal microorganisms have a critical positive effect on the maintenance of the gastrointestinal mucosa epithelium integrity, the maturation of the immune system, and the digestion and degradation of related nutrients (O’Hara et al., 2020; Peixoto et al., 2021). Comparing with the role of digesta-associated microbiota on degrading and utilizing plant fiber, the mucosal bacteria mainly participated in the maintenance of intestinal epithelial barrier integrity, which can prevent pathogen adhering or accessing to epithelial cell (Lai et al., 2022; Wu et al., 2022). It always kept in a relatively stable dynamic balance of microbiota under normal physiological status. However, under the continuous stimulation of several adverse environmental factors (such as high-grain diet) or the decrease of animal immunity, the balance of gastrointestinal microbiota will be dysbiosis, resulting in the occurrence of a variety of diseases, such as rumen acidosis, diarrhea, enteritis and so on (Parker et al., 2018; Durrant and Bhatt, 2019; Oliphant and Allen-Vercoe, 2019).

Rumen, as the major functional compartment of the digestive tract of ruminants, where inhabited by a large number of microorganisms (bacteria, archaebacteria, anaerobic fungi, and ciliate protozoa), has attracted extensive attention. Recently, goats are increasingly fed on concentrate-rich diets due to the intensification of goat production. Hindgut fermentation in goats also becomes more important with the increased flow of undigested components into hindgut (Abeyta et al., 2023). Nevertheless, compared to the rumen, there is little information regarding the fermentation processes in the hindgut. Furthermore, most studies have kept the focus on the effect of diet (Tao et al., 2017; Lin et al., 2021; Xie et al., 2021; Zhang et al., 2021), while the effect of age on the hindgut fermentation has received little attention. Several studies explored the change of gastrointestinal microbiota before or after weaning and displayed that the similarity between each age group increased and the composition of bacterial community gradually developed toward matured with goats aged (Han et al., 2015; Lei et al., 2018; Yeoman et al., 2018; Li B. et al., 2019), while another researcher assumed that although the gastrointestinal microbiota of calves just after weaning was more similar to that of adults than neonatal, there were still distinct differences compared with adult cows (Dill-McFarland et al., 2017).

Hence, this study investigated the dynamics of the bacterial community in digesta and mucosa samples of the rumen, cecum, and colon of cashmere goats at 0-, 14-, 28-, 42-, 56-days old, 4-, 6-, and 18-month-old and through comparing the spatial-temporal change difference of microbiota to clarify the colonial process of bacteria in the rumen and hindgut. This information will promote the systematic comprehension of microbiota and their biological function in gastrointestinal tracts (GITs) and provide new insight into probiotics intervention to improve animal health and production.

## Materials and methods

### Animals and sample collection

All animals in this study were handled in accordance with the guidelines of the China Council on Animal Care and the Ministry of Agriculture of the People’s Republic of China, and the sampling procedures were authorized by the Experimental Animal Manage Committee of Shanxi Agricultural University. A total of 24 neonatal kids born to 30 female goats with similar ages and body weights under the estrus synchronization protocol that proportioned an amplitude maximum of 7 days were obtained immediately after birth. Based on the sampling time, the kids were randomly distributed to 8 age groups: 0-, 14-, 28-, 42-, 56-day-old, 4-, 6-, and 18-month-old, with 3 replicates in each group. In the 0-day-old age group, newborn kids were isolated from the female goat and executed promptly before sucking milk. In other age groups, each of kids was reared together with their respective mothers and they were solely fed with colostrum from 0 to 3 days old and then suckled raw milk until 25 days of age. The kids initiated to ingest complete formula granulated feed (Supplementary Table 1) from 25 days besides breast milk. The kids were weaned after 56 days old. All kids were fed two equal portions of the diet at 8:00 and 18:00 and had free access to water throughout the whole experiment. By the respective deadline, the kids of each age group were euthanized via injection of thiopental (0.125 mg/kg of body weight) and potassium chloride (5 to 10 mL). After execution, the enterocoelia was opened and digesta (72) and mucosa (71) tissue samples were collected from the rumen, cecum, and colon. The digesta in each GIT was homogenized and then sampled to a 5 mL cryogenic vials and immediately frozen in liquid nitrogen. Meanwhile, the mucosal tissue sample was cut into approximately 2 cm × 2 cm and rinsed 3 times with sterile and cold phosphate-buffered saline (PBS) to remove the digesta and snap-frozen in liquid nitrogen. Finally, the homogenized digesta and mucosal sample from each GIT segment were stored at −80°C for subsequent DNA analysis.

### DNA abstract and 16S rRNA gene sequencing

For DNA extraction, one gram (wet weight) of homogenized digesta and mucosa sample of each GIT segment was used. The DNA was abstracted by following the procedures described by Li B. et al. (2019). The DNA concentration was quantified using a Nanodrop spectrophotometer and stored at −20°C until further processing. The V3–V4 hypervariable regions of the bacteria 16S rRNA gene were amplified in triplicate by PCR using the following procedure: 95°C for 2 min, followed by 25 cycles at 95°C for 30 s, 55°C for 30 s, and 72°C for 30 s and a final extension at 72°C for 5 min, with the primers described by Xu et al. (2016). PCR reaction mixture consisted of 4 μL of 5 × FastPfu Buffer, 2 μL of 2.5 mM dNTPs, 0.8 μL of each primer (5 μM), 0.4 μL of FastPfu Polymerase, 10 ng of template DNA and water to 20 μL. PCR products were recovered from a 2% agarose gel and purified using an AxyPrep DNA Gel Extraction Kit (Axygen Biosciences, Union City, CA, USA). Purified amplicons were pooled in equimolar and paired-end sequenced (2 × 300 bp) on an Illumina MiSeq platform according to standard protocols (Caporaso et al., 2012). Raw reads of different samples were demultiplexed and quality-filtered using default parameters in Quantitative Insights into Microbial Ecology (QIIME, version 1.9.1) (Caporaso et al., 2010). In this study, the potential contaminant genotypes (16S rDNA sequences possibly originating from reagents or instruments) were removed from the data after pre-clustering and chimera removal. The negative controls (in the form of a PCR-amplified kit blank sample) were used to control the errors. Then, valid and clean sequences were assigned to bacteria using the cluster command in Mothur 1.3.

### Bioinformatic analysis

The operational taxonomic unit (OTU) was clustered with a 97% similarity cutoff using UPARSE (version 7.1) (Edgar, 2013). The taxonomic classification was performed to the genus level and aligned to the SILVA 16S rRNA database (version 132) using a confidence threshold of 0.7 (Quast et al., 2012). Alpha (Good’s coverage, Chao, and Shannon indexes) and beta diversities were calculated for downstream analysis of OTUs via using the Mothur (1.30.2) and Qiime (1.9.1), respectively. Principal coordinate analysis (PCoA) based on the Bray-Curtis distance was applied to visualize the dissimilarity of microbial communities among different age groups. Analysis of similarity (ANOSIM) was conducted to assess significant differences among samples using Mothur version 1.3 (Schloss et al., 2009). The data of the digesta sample in the 0-, 14-, 28-, 42- and 56-day-old age groups was collected from Li B. et al. (2019) article and the accession number of SRA in NCBI was SRP195450. In this study, phylogenetic investigation of communities by reconstruction of unobserved states 2 (PICRUSt 2) was used to predict the molecular function of each sample (Langille et al., 2013). The presumable genes and their functions are pre-calculated in the database of the Kyoto Encyclopedia of Genes and Genomes (KEGG)^1^.

### Quantitative real-time PCR analysis

The copy numbers of the 16S rRNA genes related to total bacterial populations was calculated via real-time PCR, which was performed in triplicate with the SYBR Premix Ex Taq II assay kit (TaKaRa Bio Inc., Shiga, Japan) using a CFX96 Real-Time PCR Detection System (Bio-Rad Laboratories, Hertfordshire, UK). The primers for total bacteria were descripted in Metzler-Zebeli et al. (2013). The reaction mixture and protocol were in accordance with the author’s previous article (Li B. et al., 2019).

### Statistical analysis

The differences of OTU, Chao and Shannon indexes, the variance of relative abundance of bacterial taxa, and the total copy number of bacterial 16S rRNA gene affected by ages and GIT regions were analyzed using SPSS (SPSS v.20, SPSS Inc., Chicago, IL, USA) via two-way ANOVA with Tukey’s *post hoc* comparison. One-way ANOVA was adopted to compare the relative abundance values of KEGG pathways among age groups within a region or GIT regions. The comparison of these indices between in digesta and mucosa were analyzed via *T*-test. Significance was declared at *P* < 0.05.

## Results

### Data acquisition and analysis

Through the 16S rRNA gene sequencing for 143 samples collected from the digesta and mucosa of the rumen, cecum and colon, a total of 5,138,292 valid reads were acquired with an average of (35,932 ± 578) reads per sample. The reads of all samples were subsampled to 19,816 based on the minimum sequence to facilitate unified analysis (Supplementary Table 2). The individual-based refraction curves entered a platform stage, which reflected the sequencing depth was sufficient to accurately describe the bacterial composition of each sample (Supplementary Figure 1). And the result was confirmed by the Good’s coverage, of which all sample exceeded 0.98. The whole number of OTUs reached 5,859 according to a specialized criterion (≥ 97% nucleotide sequence identity between reads). A total of 1,003 genera, or 487 families, or 258 orders, or 99 classes belonging to 43 phyla were identified from all samples.

### Digesta-associated bacterial diversity and composition in the rumen, cecum, and colon

**FIGURE 1:**
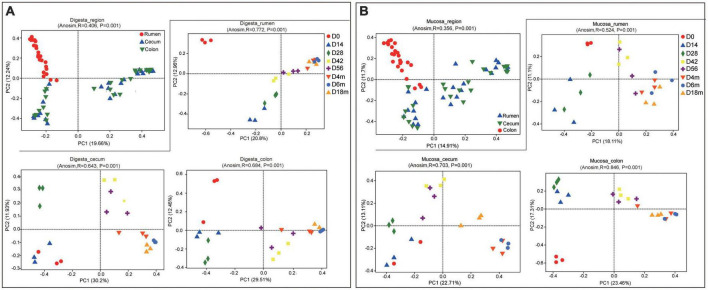
Principal coordinate analysis (PCoA) profile of microbial composition in rumen, cecum and colon (based on Bray-Curtis dissimilarity metric). **(A)** PCoA of bacterial microbiota in sample of digesta according to age group in each GIT region. **(B)** PCoA of bacterial microbiota in sample of mucosa according to age group in each GIT region.

**FIGURE 2:**
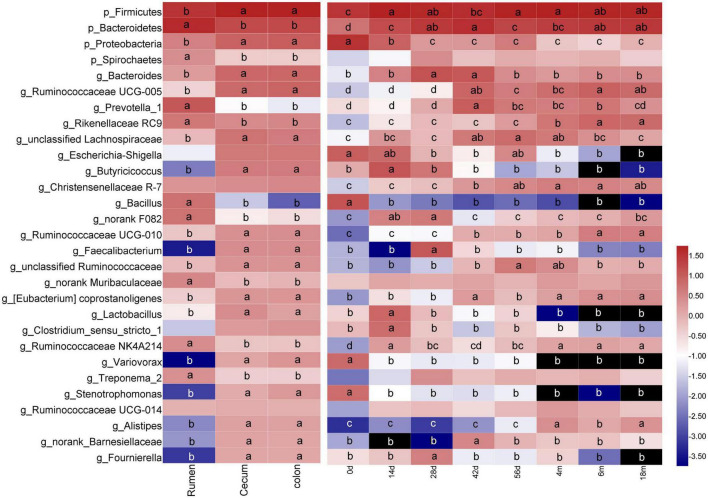
Changes of common dominant bacterial taxa (sequences summarized at phylum [p_] and genus [g_] levels at average abundance of ≥1%) in sample of digesta according to GIT region or age group. Means followed by the different letter are significantly different (*P* < 0.05). Taxon rows followed by the same letter or without letters were not significantly different for that variable (*P* > 0.05).

In digesta, both the GIT and age had a significant influence on bacterial richness and diversity. Also, there was a significant interaction between GIT and age on the relative richness and diversity of bacteria (*P* < 0.001 to *P* < 0.05). The bacterial OTU, Chao and Shannon in rumen were notably higher than these in cecum and colon (*P* < 0.05). In general, the relative richness and diversity of bacterial community increased markedly with the goats aged other than decreased slightly at 14 days of age (Supplementary Table 3). Based on the analysis of PCoA (using Bray-Curtis distance) and ANOSIM, the bacterial community composition in digesta of the rumen differed obviously from that in the cecum and colon, while there was a higher similarity in bacterial community composition between in cecum and colon (ANOSIM, *r* = 0.406, *P* = 0.001). In the rumen, compared to 0-day-old and 14-day-old groups, samples from age groups after 28 days were basically clustered together, which indicated that rumen bacterial microbiota may not initiate to develop into a stable structure until the age of 28 days. In the cecum, the samples clustered separately among each age group except for the 0- vs. 14-day age groups, 42-vs. 56-day age groups, and the 4-, 6- vs. 18-months age groups; in colon, there was only higher similarity between the 14- and 28-day age group, 42- and 56-day age group, and 4-, 6-, and 18-months age group (Figure 1A and Supplementary Table 4). These results indicated that in in the digesta of rumen, cecum and colon, the bacterial community showed obvious age effect and the distance of samples within or between age groups decreased gradually, suggesting the similarity of bacterial composition increased with goats aged. In digesta samples of the rumen, cecum and colon, a total of 35 phyla were identified (Supplementary Figure 2A). With performing Venn diagram analysis, 24 phyla were found to be shared in the rumen, cecum, and colon (Supplementary Figure 2B). The majority of these shared taxa belong to Firmicutes (64.03%), Bacteroidetes (20.44%), and Proteobacteria (10.99%) (Supplementary Figure 2C). In the rumen, the phylum Firmicutes dominated all microbiota at birth and then was replaced by Bacteroidetes after 14 days of age. In the cecum and colon, Proteobacteria, the predominant phylum, was displaced by Firmicutes with goat aged. The proportion of Bacteroidetes and Spirochaetes in the rumen was significantly higher than these in the cecum and colon, while Firmicutes and Proteobacteria were higher in the cecum and colon (*P* < 0.05) (Figure 2).

**FIGURE 3:**
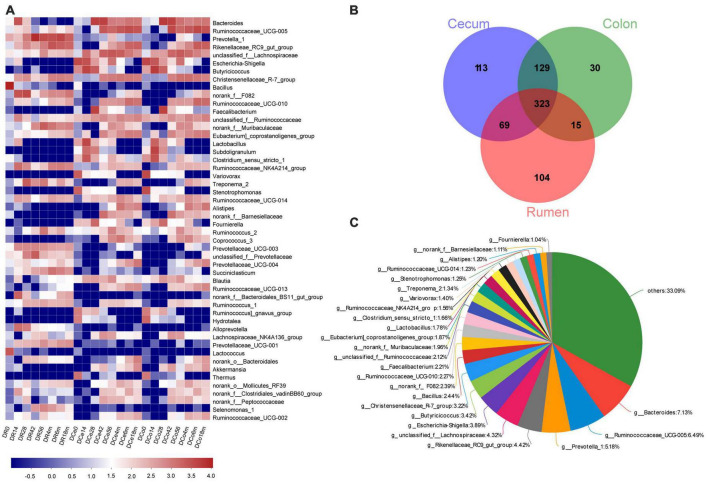
Bacterial community composition of digesta sample at the genus level. **(A)** Bacterial taxa composition in rumen, cecum and colon at different age (the top 50 genera). **(B)** Venn diagram of bacterial genera shared between rumen, cecum, and colon. **(C)** Relative proportion of predominant common genera (those with an average proportion ≥1% based on all common genera).

At the genus level, 783 genera were found in digesta samples of rumen and hindgut. Among the top 50 genera, the genera *Bacillus*, *Prevotellaceae UCG-003*, unclassified_f_Prevotellaceae, *norank_f_Bacteroidales_BS11_gut_group*, *Alloprevotella*, *Prevotellaceae_UCG-001*, *Lactococcus* and *Selenomonas_1* was predominant in the rumen, while in cecum and colon, there were mainly consisted of *Escherichia-Shigella*, *Butyricicoccus*, *Faecalibacterium*, *Subdoligranulum*, *Variovorax*, *Stenotrophomonas*, *Alistipes*, *Fournierella*, *Coprococcus_3*, *Hydrotalea*, *Akkermansia* and *Thermus* (Figure 3A). A total of 323 genera were identified to be shared among the digesta samples of rumen, cecum and colon by performing Venn diagram analysis (Figure 3B). There were 25 core genera (those with the average proportion ≥1% based on all common genera) among these common genera (Figure 3C). The majority of these core genera varied notably according to GIT region, age or GIT region and age. The genera *Prevotella_1*, *Rikenellaceae RC9*, *Bacillus*, *norank F082*, *norank Muribaculaceae*, *Ruminococcaceae NK4A214*, and *Treponema_2* in rumen were higher remarkably than in cecum and colon, but the genera *Bacteroides*, *Ruminococcaceae UCG-005*, unclassified Lachnospiraceae, *Escherichia-Shigella*, *Butyricicoccus*, *Ruminococcaceae UCG-010*, *Faecalibacterium*, unclassified Ruminococcaceae, *Lactobacillus*, *Variovorax*, *Stenotrophomonas*, *Alistipes*, *norank_Barnesiellaceae*, and *Fournierella* were enriched in cecum and colon (*P* < 0.05). In addition, a total of 22 genera varied significantly exclusively ascribed to goat age. The genera *Bacillus*, *Escherichia-Shigella*, *Variovorax*, and *Stenotrophomonas* were highest at 0 days and then decreased extremely as the goat aged. Several taxa including *Bacteroides*, *Prevotella_1*, unclassified Lachnospiraceae, *Butyricicoccus*, *norank_f_F082*, *Faecalibacterium*, *Lactobacillus*, and *Clostridium sensu stricto 1* increased notably firstly then decreased markedly with increasing age increasing. And the genera *Ruminococcaceae UCG-005*, *Rikenellaceae RC9*, *Christensenellaceae R-7*, *Ruminococcaceae UCG-010*, *Ruminococcaceae NK4A214*, and *Alistipes* increased significantly as goat aged (*P* < 0.05) (Figure 2 and Supplementary Table 5).

Furthermore, the GIT region and age had simultaneously a significant influence on several bacterial taxa. In the rumen, the genus *Bacillus* was highly enriched in the 0-day-old group than in other age groups (*P* < 0.05). By contrast, the genera *Bacteroides*, *norank F082*, *Ruminococcaceae NK4A214*, and *Treponema 2* increased significantly in 14- or 28-day group, while the genera *Prevotella 1* and *Rikenellaceae RC9* increased notably as goats aged (*P* < 0.05). The major changes observed in the cecum and colon included increases in the *Bacteroides*, *Butyricicoccus*, *Faecalibacterium*, and *Lactobacillus* in 14-, 28-, or 42-day group (*P* < 0.05), the *Ruminococcaceae UCG-005*, *Ruminococcaceae UCG-010*, and *Alistipes* as goats aged (*P* < 0.05), and decreases in *Escherichia-Shigella*, *Variovorax*, and *Stenotrophomonas* with increasing age (*P* < 0.05) (Supplementary Table 5).

The quantitative result of the copy number of total bacteria performed via real-time PCR showed that during the pre-weaning period, the total bacterial population in the rumen was higher than that in the cecum and colon; however, it was given an opposite result after weaning. Generally, the copy number of total bacteria increased significantly as goats aged (*P* < 0.05) (Table 1).

**TABLE 1:** The total bacterial number [log10 (gene copies)/g sample] in digesta and mucosa sample of rumen, cecum and colon.

	Age									SEM		*P*	
GIT site	0 day	14 day	28 day	42 day	56 day	4 month	6 month	18 month	G		G	A	G × A
**Digesta**													
Rumen	–	10.217^A^	9.932	10.618	10.703^A^	10.867	10.649	9.655^B^	10.377^A^	0.082	0.001	<0.001	0.019
Cecum	5.919^d^	8.481^Bc^	9.893^abc^	10.393^ab^	9.139^Bbc^	10.796^a^	10.838^a^	10.722^ABa^	9.522^B^				
Colon	5.539^e^	9.634^ABcd^	10.434^bc^	10.377^bc^	9.385^Bd^	10.818^ab^	10.955^ab^	11.583^Aa^	9.841^B^				
A	5.729^c^	9.444^b^	10.087^ab^	10.463^a^	9.742^b^	10.827^a^	10.814^a^	10.653^a^					
**Mucosa**													
Rumen	6.846^c^	7.884^bc^	8.010^bc^	8.112^abc^	8.110^abc^	9.453^a^	9.235^ab^	9.225^ab^	8.359^A^	0.069	0.029	<0.001	0.097
Cecum	6.939	7.453	8.320	7.778	8.315	8.5145	7.832	7.931	7.899^B^				
Colon	7.020^b^	8.059^a^	8.176^a^	8.462^a^	8.382^a^	7.907^a^	8.564^a^	8.445^a^	8.127^AB^				
A	6.935^b^	7.799^ab^	8.169^a^	8.118^a^	8.269^a^	8.635^a^	8.544^a^	8.534^a^					

(1) Mean values with different superscripted lowercase letters within the same row differ significantly (P < 0.05) while with different superscripted capital letters within the same column means differ significantly (P < 0.05). (2) A means ages, GIT means gastrointestinal tract. (3) SEM, standard error of the difference of the means. The same as below in Table 2.

### Mucosa-associated bacterial diversity and composition in the rumen, cecum, and colon

As for the mucosal sample, the age had a significant effect on bacterial richness and diversity (*P* < 0.05). Nevertheless, there was no notable effect of the GIT region on bacterial richness and diversity. Whether in the rumen, cecum, or colon, the OTU, Chao and Shannon of bacterial community decreased significantly in 14-day group and then increased markedly as goats aged (*P* < 0.05). Additionally, the indices mentioned above were lowest in the rumen and highest in colon (Supplementary Table 3). Consistent with the sample of digesta, a significant difference in composition and structure of the bacterial community between in the ruminal mucosa and in the cecal or colonic mucosa was revealed, and it didn’t separate clearly between in the cecum and colon. In ruminal mucosa, the samples clustered separately among each age group with the exception of 14- vs. 28-day age groups, 56-day, 4-, 6- vs. 18-month age groups. In the cecum, the samples clustered closely between 0-day and 14-day age group, 42-day and 56-day age group, and 4-month and 6- month-old age group; In colon, the samples separated spatially among each age group other than in 14- vs. 28-day group, 42- vs. 56-day age group, and 4-, 6- vs. 18-month age group (Figure 1B). All of results indicated the similarity of bacterial composition increased with goats aged.

At the phylum level, a total of 43 taxa were obtained from the mucosal sample of rumen, cecum and colon, and there were 37 phyla that coexisted in these regions through Venn diagram analysis (Supplementary Figures 3A, B). The phylum Firmicutes (42.47%), Bacteroidetes (25.10%), Proteobacteria (13.27%), Spirochaetes (11.03%), Epsilonbacteraeota (2.91%), and Patescibacteria (1.01%) dominated among these shared taxa (Supplementary Figure 3C). The phylum Firmicutes and Spirochaetes were enriched significantly in the cecum and colon than in the rumen, and the Bacteroidetes, Proteobacteria, and Epsilonbacteraeota were mainly presented in the rumen (*P* < 0.05). In the rumen, the Firmicutes and Bacteroidetes increased signally as goats aged; while in the cecum and colon, these two phyla increased, observed firstly and then decreased with increasing age (*P* < 0.05). Wherever the Proteobacteria increased significantly as goats aged. The Spirochaetes were dominant in older age groups (4-, 6- and 18-month age groups) in the cecum and colon (Supplementary Figure 5).

A total of 957 genera were found in these three GIT tissues. The top 50 genera inhabited in the mucosal sample of the rumen, cecum and colon were displayed in Supplementary Figure 4A. With the analysis of Venn diagram, there were 718 shared genera among the sample of the rumen, cecum and colon. The number of bacteria shared between the cecum and colon (823) was much higher than that shared with rumen (731 or 759), respectively, which implied that the similarity of bacterial community between in cecum and colon was rather higher (Supplementary Figure 4B). A total of 21 predominant genera was found among these shared genera (Supplementary Figure 4C). And most genera varied significantly according to different GIT regions and/or ages. The genera *Butyrivibrio 2*, *Prevotella 1*, *Prevotellaceae UCG-001*, *Campylobacter*, *norank_f_Neisseriaceae*, unclassified_f_Pasteurellaceae and *norank_f_F082* were mainly enriched in the rumen, while the genera *Treponema 2*, *Bacteroides*, *Escherichia-Shigella*, unclassified_f_Lachnospiraceae, *Ruminococcaceae UCG-005*, *Ruminococcaceae UCG-010*, *Butyricicoccus*, *Faecalibacterium*, *Subdoligranulum* and unclassified_f_Ruminococcaceae were higher in cecum and colon than these in rumen (*P* < 0.05). A total of 16 genera changed remarkably as goat aged (Supplementary Figure 5). Moreover, the GIT region and age had significant effects on several genera simultaneously. In the rumen, the genera *Butyrivibrio 2*, *Prevotella 1*, and *Prevotellaceae UCG-001* increased notably as goat aged, while the genera *Campylobacter*, *norank_f_Neisseriaceae*, and *norank_f_F082* increased significantly in 14- or 28-days-old age group (*P* < 0.05). The unclassified_f_Pasteurellaceae was mainly enriched in 0-day-old group. The major changes observed in the cecum and colon included increases in the *Bacteroides*, *Butyricicoccus*, *Faecalibacterium*, and *Subdoligranulum* in 14- or 28-day-old age group, the unclassified_f_Lachnospiraceae and *Ruminococcaceae UCG-005* in 42- or 56-days-old group, the *Treponema 2* and *Ruminococcaceae UCG-010* as goat aged, and decreases in *Escherichia-Shigella* with increasing age (*P* < 0.05) (Supplementary Figure 5 and Supplementary Table 6).

Quantitative results of the rumen, cecum and colon showed that the copy number of total bacteria in a mucosal sample of the rumen was slightly less than that in the cecum and colon before weaning. But after weaning, it was highest in the rumen. In rumen, the total bacterial population increased significantly as the goat aged. Generally, the total number of bacteria in the mucosal sample of GIT was not stable until 28-day old. Compared to neonatal kids, it was higher significant in other older age groups (*P* < 0.05), but there was no significant difference between each other (Table 1).

### Comparison of digesta- and mucosa-associated bacterial microbiome in rumen, cecum, and colon

In the rumen, the Shannon of the bacterial community in digesta was significantly higher than in the mucosa (*P* < 0.05), and there was no significant difference in OTU and Chao between digesta and mucosa. In the cecum, there were no significant differences in OTU, Chao and Shannon values between the digestive and mucosal microbiota, but the Chao and the number of OTU were slightly higher in mucosa. In colon, the Chao and the number of OTU in the mucosa were significantly higher than in digesta (Supplementary Figure 6). Based on the PCoA analysis, there was a clear distinction between the samples of digesta and mucosa in rumen (ANOSIM, *r* = 0.274, *P* = 0.001), which indicated that the composition and structure of bacterial community between in digesta and mucosa differed apparently. In the cecum and colon, the sample of digesta and mucosa in each age group clustered closely during the pre-weaning period, i.e., they came together in clusters with age, which represented there was a certain age correlation. After weaning, the samples of digesta and mucosa clustered separately whatever at each age (Supplementary Figure 7). All of these implied that in the cecum and colon, the similarity of the bacterial community in the sample of digesta and mucosa decreased gradually and played different physiological functions as goat aged.

#### Comparison of digesta- and mucosa-associated microbiota in rumen

**FIGURE 4:**
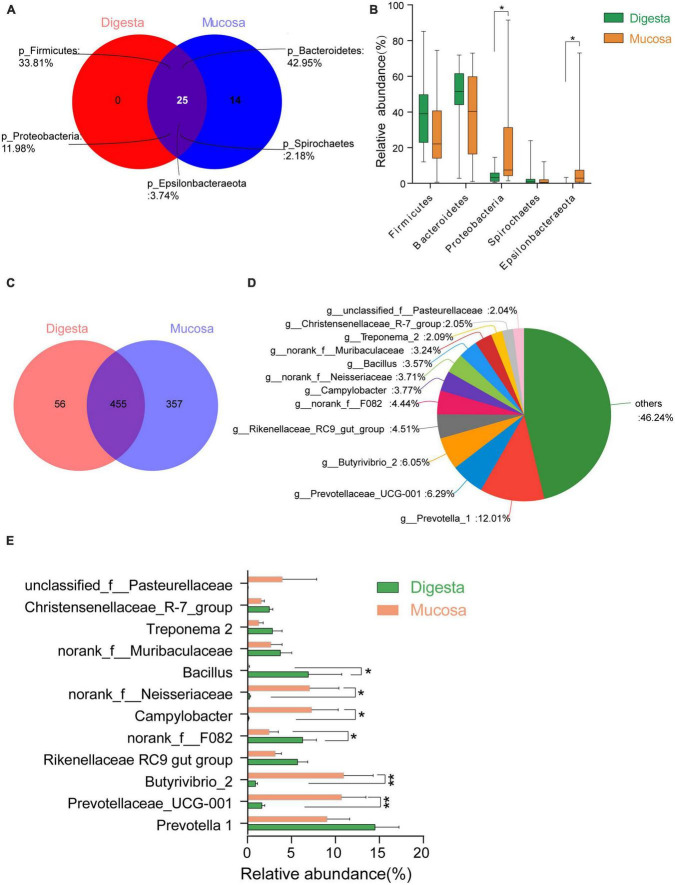
Comparison of bacterial taxa between in digesta and mucosa sample of rumen. **(A)** Venn diagram of bacterial phyla shared between in sample of digesta and mucosa sample. **(B)** Comparison of common predominant bacterial phyla between digesta and mucosa sample (those with an average proportion ≥1%). **(C)** Venn diagram of bacterial genera shared between in digesta and mucosa sample. **(D)** Predominant genera composition shared between digesta and mucosa sample (those with an average proportion ≥2%). **(E)** Comparison of common predominant bacterial genera between digesta and mucosa sample. Bars with a star symbol above their whiskers are significantly different between digesta and mucosa sample using *T*-test analysis; “*” means “0.01 < *P* < 0.05,” “**” means “0.001 < *P* < 0.01.”

A total of 39 phyla were found in the rumen. There were 25 shared phyla between digesta and mucosa, and 14 unique phyla were attributable to the mucosa. Among these shared genera, the majority of sequences belonged to Firmicutes, Bacteroidetes, Proteobacteria, Epsilonbacteraeota and Spirochaetes. The phyla Proteobacteria and Epsilonbacteraeota in the mucosa were significantly higher than in digesta, but the proportion of Firmicutes and Bacteroidetes were higher in digesta (*P* < 0.05) (Figures 4A, B). As for the genus level, 868 genera were identified and out of 455 genera were shared between in digesta and mucosa. Twelve dominant genera were found in these common genera (Figures 4C, D). The genera *Bacillus* and *norank_f_F082* in digesta were higher markedly than in mucosa, while the genera *Prevotellaceae UCG-001*, *Butyrivibrio 2*, *Campylobacter*, and *norank_f_Neisseriaceae* were mainly enriched in the mucosa (*P* < 0.05) (Figure 4E).

#### Comparison of digesta- and mucosa-associated microbiota in cecum

A total of 902 genera assigned to 40 phyla were found in the sample of the cecum. Thirty-two phyla were shared between in digesta and mucosa, with 2 unique taxa in digesta and 6 unique taxa in the mucosa (Supplementary Figure 8A). The phyla Firmicutes, Bacteroidetes, Proteobacteria and Spirochaetes dominated all shared bacterial communities. Thereinto, the proportion of Firmicutes in digesta was higher significantly than in mucosa, while the Spirochaetes was higher in the mucosa (*P* < 0.05) (Supplementary Figure 8B). At genus level, there were 583 common genera between in digesta and mucosa and a core microbiome composed of 14 bacterial taxa were identified in these shared genera (Supplementary Figures 8C, D). The genus *Treponema 2* was more enriched in digesta compared to the mucosa, and unclassified Ruminococcaceae was higher in the sample of digesta (*P* < 0.05). In addition, the proportion of *Ruminococcaceae UCG-005*, *Butyricicoccus*, *Faecalibacterium*, and *Lactobacillus* were a little higher in digesta, and the genera *Escherichia-Shigella* and *Clostridium sensu stricto 1* occupied a higher percent in the mucosa (Supplementary Figure 8E).

#### Comparison of digesta- and mucosa-associated microbiota in colon

A total of 915 genera belonging to 43 phyla was identified in the colon, in which 27 phyla were shared between in digesta and mucosa, with one and 15 unique phyla ascribed to digesta and mucosa, respectively (Supplementary Figure 9A). Among the shared taxa, also the phyla Firmicutes, Bacteroidetes, Proteobacteria and Spirochaetes were predominant. The Firmicutes in digesta was higher significantly than in mucosa, while the Spirochaetes was more enriched in the mucosa (*P* < 0.05), which corresponded with that in the cecum (Supplementary Figure 9B). Additionally, 471 shared genera were found in samples of digesta and mucosa, in which 13 dominant genera were identified (Supplementary Figures 9C, D). The genus *Ruminococcaceae UCG-005* was more enriched in digesta, while the *Treponema 2* in the mucosa was higher significantly in mucosa than in digesta (*P* < 0.05) (Supplementary Figure 9E).

### Predicted bacterial function based on 16S rRNA gene sequencing

**FIGURE 5:**
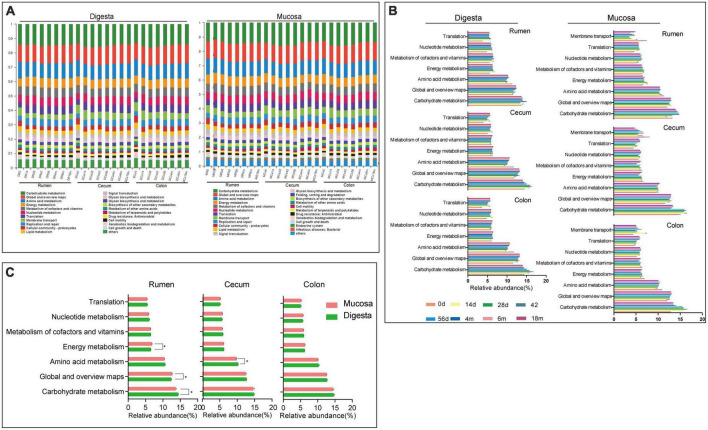
16S rRNA gene functional predictions. **(A)** Variations in KEGG metabolic pathways of microbiota at different ages in digesta and mucosa sample of rumen, cecum and colon. **(B)** Changes of the predominant KEGG metabolic pathways in each age group. **(C)** Comparison of KEGG metabolic pathways of microbiota between digesta and mucosa sample in rumen, cecum, and colon.

As a predictive exploratory, PICRUSt2 was used to probe the molecular function of the bacterial community in the sample of the rumen and hindgut. At the KEGG level 2, a total of 39 KEGG pathways were found in digesta and mucosa of the rumen, cecum and colon (Figure 5A). Among these pathways, the overwhelming majority of sequences were assigned to Carbohydrate metabolism (14.58% in digesta, 14.21% in mucosa), Global and overview maps (12.56, 12.54%), Amino acid metabolism (10.35, 10.08%), Energy metabolism (6.29, 6.37%), Metabolism of cofactors and vitamins (6.10, 6.00%), Nucleotide metabolism (5.77, 5.79%), and Translation (5.19, 5.22%). In digesta, the proportion of gene families associated with Nucleotide metabolism and Translation in the rumen were significantly higher than in the cecum and colon, while the genes involved in Global and overview maps were more enriched in the cecum and colon (*P* < 0.05). In mucosa, the relative abundance of genes related to Carbohydrate metabolism and Membrane transport in the cecum and colon were higher than in the rumen (*P* < 0.05). Whatever, the proportion of genes involved in Amino acid metabolism, Energy metabolism and Metabolism of cofactors and vitamins were more enriched in the rumen (*P* < 0.05) (Table 2). Moreover, in digesta sample of rumen, cecum and colon, the abundance of genes associated with Carbohydrate metabolism increased firstly then decreased as the goat aged, but there was an opposite trend on the gene involved in Amino acid metabolism. Compared to other age groups, the proportion of genes related to Nucleotide metabolism and Translation was lowest in the 0-day-old age group. In the mucosal sample, a similar trend was observed in the change of the genes linked to Carbohydrate metabolism compared to in digesta, while the proportion of genes involved in Nucleotide metabolism and Translation increased significantly as goat aged (Figure 5B). In the rumen, the relative abundance of genes linked to Carbohydrate metabolism in digesta was higher notably than in mucosa, while the genes involved in Energy metabolism and Global and overview maps were more enriched in the mucosa (*P* < 0.05). In the cecum, the abundance of genes related to Amino acid metabolism was higher in digesta compared to in mucosa (*P* < 0.05) (Figure 5C).

**TABLE 2:** Comparison of predominant bacterial function among rumen, cecum and colon (average proportion ≥ 5%).

Items	Rumen	Cecum	Colon	SEM	*P*
**Digesta**					
Carbohydrate metabolism	14.367	14.739	14.630	0.128	0.118
Global and overview maps	12.375^b^	12.634^a^	12.660^a^	0.066	0.006
Amino acid metabolism	10.568^a^	10.207^b^	10.268^b^	0.103	0.037
Energy metabolism	6.493^a^	6.205^b^	6.178^b^	0.026	<0.001
Metabolism of cofactors and vitamins	6.459^a^	5.923^b^	5.920^b^	0.045	<0.001
Nucleotide metabolism	6.038^a^	5.651^b^	5.611^b^	0.057	<0.001
Translation	5.453^a^	5.079^b^	5.044^b^	0.063	<0.001
**Mucosa**					
Carbohydrate metabolism	13.631^b^	14.612^a^	14.411^a^	0.116	<0.001
Global and overview maps	12.607	12.446	12.549	0.086	0.242
Amino acid metabolism	10.362^a^	9.813^b^	10.039^b^	0.066	0.002
Energy metabolism	6.787^a^	6.089^b^	6.214^b^	0.070	<0.001
Metabolism of cofactors and vitamins	6.418^a^	5.732^b^	5.850^b^	0.052	<0.001
Nucleotide metabolism	5.874	5.780	5.723	0.052	0.120
Translation	5.380^a^	5.132^b^	5.146^b^	0.069	0.016
Membrane transport	4.320^b^	5.559^a^	5.183^a^	0.180	<0.001

Mean values with different superscripted lowercase letters within the same row differ significantly (P < 0.05), while the mean values with no letters or with the same superscripted lowercase letters means no significant difference.

At the KEGG level 3, a total of 13 predominant pathways was identified in digesta and mucosa of rumen, cecum and colon (Supplementary Table 7). Whatever in sample of digesta or mucosa, the proportion of sequences associated with Purine metabolism (ko00230) and Oxidative phosphorylation (ko00190) in rumen were markedly higher than in cecum and colon, while the genes related to ABC transporters (ko02010), Quorum sensing (ko02024), Two-component system (ko02020), and Pyruvate metabolism (ko00620) were mainly enriched in cecum and colon (*P* < 0.05) (Supplementary Table 8). Furthermore, the age had a significant influence on the abundance of predominant KEGG pathways. The proportion of genes related to Two-component system (ko02020) was higest in 0-day-old age group (*P* < 0.05), while the genes associated with Biosynthesis of amino acids (ko01230) mainly enriched in older age groups (*P* < 0.05). The abundance of sequences related to Glycolysis/Gluconeogenesis (ko00010) and Pyruvate metabolism (ko00620) were higher significantly in 28- or 42-day-old age group than other age groups (*P* < 0.05) (Supplementary Table 9). In rumen, the proportion of the genes related to Glycolysis/Gluconeogenesis (ko00010) in sample of digesta was higher significantly than in mucosa (*P* < 0.05). In cecum, the sequences involved in Carbon metabolism (ko01200) was also more enriched in digesta (*P* < 0.05) (Supplementary Table 10).

## Discussion

The object of this experiment was to study the dynamics of the bacterial community in digesta and mucosa sample of rumen, cecum, and colon of cashmere goats (from birth to adult) to enable a better dietary intervention to improve animal health and performance. Alpha diversity results indicated whatever digesta or mucosa sample, the number of OTU, Chao and Shannon of the bacterial community in the rumen, cecum and colon decreased significantly at 14 days of age, and then increased significantly with the goats aged. Another two studies regarding the dynamic variation of the ruminal bacterial community in goats or calves from birth to adult showed that the OTU number, relative richness, and diversity of bacterial community increased notably with aged (Jami et al., 2013; Wang et al., 2016), which was slightly different from this experiment. This may be attributed to different sampling time points or treatment methods. In this study, for 0-day-old age group, the kids were isolated from the female goat shortly before sucking milk and then the sample was collected. It is given that many microorganisms colonized rapidly into GIT in the first hours of life, the majority of which were mainly derived from the mother’s vagina or skin. These maternally derived microorganisms play a critical role in the establishment and development of gastrointestinal microbiota in young animals (Yeoman et al., 2018; Zhang et al., 2019). Breast milk (especially colostrum) is not only a source of gastrointestinal microbes for kids but also contains a variety of nutrients and immunoglobulins, which can inhibit harmful bacteria and selectively shape the colonization of gastrointestinal microbiota (Ruiz et al., 2014). This may be one of the reasons that the abundance and diversity of the bacterial community in the rumen, cecum and colon decreased significantly at 14 days of age. In this study, the abundance of the bacterial community in rumen digesta was higher significantly than in the cecum and colon, while in mucosa, the relative richness and diversity of bacteria were highest in the colon and lowest in the rumen. In addition, the Shannon of the bacterial community in ruminal digesta was higher significantly than that in its mucosa, while the OTU number and Chao of microbiota in colonic mucosa was higher than in its digesta. Results of qPCR showed that in digesta sample, the copy number of total bacteria in rumen was higher than in cecum and colon in pre-weaning goats, while it was lower in rumen after weaning; as for in the mucosal sample, compared with in the cecum and colon, the copy number of total bacteria was lower and higher in rumen before and after weaning, respectively. In a study on the gastrointestinal microbiota of adult dairy cattle, the abundance and diversity of the bacterial community in the sample of digesta and mucosa were highest in the rumen compared to the cecum and colon. The copy number of total bacteria in the sample of digesta in the rumen was lower than in the cecum and colon, while in the mucosal sample, it was higher in the rumen. The distribution of the copy number of total bacteria was basically consistent with this experiment, but the abundance and diversity index of the bacterial community in the mucosa were different (Mao et al., 2015). Another study claimed that the abundance of the bacterial community in the mucosal sample of the rumen, cecum and colon was higher than in respective digesta and the abundance of the bacterial community in the mucosa was highest in the rumen (Malmuthuge et al., 2014), which was inconsistent with this experiment. This may be attributed to the different experiment objects or sampling time points. PCoA and ANOSIM results indicated whatever in digesta or mucosa, the bacterial composition in the rumen separated clearly from in the cecum and colon, implying there was a spatial heterogeneity. Moreover, in any GIT region or any sample type, the microbial composition displayed a particular age characteristic; both the between-age-group and within-age-group similarity increase with age, demonstrating a convergence toward a stable and mature adult composition. These findings were verified by several previous studies (Dias et al., 2018; Li B. et al., 2019; Zhang et al., 2019). In the present experiment, there was a clear difference on the composition of microbiota between in sample of digesta and mucosa in rumen; but in the cecum and colon, a high similarity of bacterial structure and composition between in these two sample types at a certain age was observed during pre-weaning period, and after weaning, the difference of the bacterial community between in the digesta and mucosa increased gradually and played different physiological functions as goat aged.

Consistent with other studies, the phyla Firmicutes, Bacteroidetes and Proteobacteria dominated all microbiota in the sample of digesta along the GIT (Kim et al., 2019; Xue et al., 2020; Zou et al., 2020). These three bacterial taxa exhibited evident temporal-spatial specificity. In the rumen, the phylum Firmicutes, which was the first predominant taxa in newborn kids, was replaced by Bacteroidetes at 14 days; while the predominant bacterial phyla in cecum and colon changed from Proteobacteria to Firmicutes as aged. A total of 25 core genera were found in the digesta sample of the rumen, cecum, and colon. Whatever in the rumen or hindgut, these bacteria can be classified into three types: the leading, transitional, and mature taxa. And the composition of each type of bacteria varies with different GIT sites. In the rumen, the leading bacteria mainly consisted of *Bacillus* and *Lactococcus*, while the genus *Bacteroides*, *Ruminococcaceae NK4A214*, and *Treponema 2* belonged to the transitional bacteria, and the mature taxa mainly included *Prevotella 1* and *Rikenellaceae RC9*. But in the cecum and colon, the genera *Escherichia-Shigella*, *Variovorax*, and *Stenotrophomonas* were the primary leading bacteria, the transitional bacteria contained *Bacteroides*, *Butyricicoccus*, *Faecalibacterium* and *Lactobacillus*, and the genera *Ruminococcaceae UCG-005*, *Ruminococcaceae UCG-010*, and *Alistipes* belonged to the mature bacteria. Studies demonstrated that the bacteria in neonatal GITs mainly derive from the maternal vagina, skin or the surrounding environment (Yeoman et al., 2018). The proportion of transitional bacteria, such as *Bacteroides*, *Lactobacillus* and etc., which can utilize absorbable small nutrient molecule (monosaccharide, lactose, or butterfat) in milk, increased gradually with the ingestion of breast milk (especially colostrum). The acetic acid produced by these transitional bacterial taxa can be converted to butyrate by the genera *Butyricicoccus* and *Faecalibacterium*, and further to promote the development of animal’s gastrointestinal epithelium (Geirnaert et al., 2014; Dill-McFarland et al., 2016). Therefore, these two bacterial genera were positively correlated with the ingestion of breast milk. Then, with the increase of solid particles and roughage, the transitional bacterial taxa were gradually replaced by the genera which can degrade polysaccharides (amylum or cellulose), such as *Prevotella 1*, *Rikenellaceae RC9*, *Ruminococcaceae UCG-005*, *and Ruminococcaceae UCG-010* (Kim et al., 2019).

Compared to the bacterial community in the digesta of GITs, the bacteria inhabited in mucosa play a more critical role due to its close contact with to host. However, there are few studies on the composition of gastrointestinal mucosal microflora in ruminants, mainly in calves. It is given that there are significant differences in the composition of microbiota between in digesta and mucosa in calves (Malmuthuge et al., 2012, 2014). In the experiment, the phylum Spirochaetes was also the dominant taxa other than Firmicutes, Bacteroidetes and Proteobacteria. The phylum Proteobacteria was superseded by Firmicutes and Bacteroidetes as goats aged, and the phylum Spirochaetes mainly presented in the cecum and colon after weaning. There were 21 core mucosa-associated genera among rumen, cecum and colon. Similar to digesta-associated microbiota, the bacterial community in mucosa displayed particular spatial specificity. in other words, there was a significant difference between rumen and hindgut. Moreover, the colonization process of the microbiota in the mucosa can also be divided into three stages: initial, transit, and stable phase. It was worth noting that the genus *Butyrivibrio 2* was predominant in the mucosa of the rumen as goats aged. In the cecum and colon, the genus *Treponema 2* dominated all mucosal bacterial taxa. As one of the main butyrate producers, the genus *Butyrivibrio 2* can release butyrate, which is absorbed directly by the host’s gastrointestinal mucosal epithelium, enhance the utilization efficiency of butyrate, and play a vital role in the proliferation and development of rumen epithelial cells (Mao et al., 2015; Wang et al., 2017). The genus *Treponema 2*, as the principal representative taxa of Spirochaetes in the hindgut, had a disadvantageous effect on animal health. Several studies suggested that it was associated with ulcerative mastitis in dairy cattle, or laminitis in calves and sheep (Giacani et al., 2013; Karlsson et al., 2014). But other evidence indicated that the genus Treponema is a kind of fiber-degrading bacteria (Liu et al., 2016).

In this study, the diversity of the bacterial community in the mucosa was higher significant than that in digesta whatever in the rumen or hindgut. The genera *Prevotella 1*, *Rikenellaceae RC9*, *Bacillus*, *Lactobacillus*, *Butyricicoccus*, *Faecalibacterium*, *unclassified Ruminococcaceae*, and *Ruminococcaceae UCG-005* mainly presented in the sample of digesta, while in the sample of the mucosa, the dominant genera consisted of *Butyrivibrio 2*, *Prevotellaceae UCG-001*, *norank_f_Neisseriaceae*, and *Treponema 2*. It is revealed that the majority of a digesta-associated bacterial genera belong to carbohydrate degrading taxa, which can utilize mono- or polysaccharides in the diet selectively. Hence, these bacteria may play an important role in promoting the degradation and digestion of feed for the host. As for the bacterial genera in mucosa, on one hand, they can compete ecological niches with pathogenic bacteria, inhibit the adhesion of them to gastrointestinal epithelium, and further to preserve the integrity of the epithelial mucosal barrier; on the other hand, several mucosal bacteria are essential for the epithelial cell proliferation and differentiation via their metabolic products, for example, the butyrate (Huws et al., 2018; Parker et al., 2018; Oliphant and Allen-Vercoe, 2019).

It is well known that microbiota in GITs has multiple and important roles in the host’s wellbeing and proper function, such as degrading cellulose in feed, synthesizing essential amino acids and vitamins, and so on. In the present study, at the KEGG level 2, the most abundant functional pathways were gene categories associated with Carbohydrate metabolism, Global and overview maps, Amino acid metabolism, Energy metabolism, Metabolism of cofactors and vitamins, Nucleotide metabolism, and Translation. These dominant functional pathways represent significant spatiotemporal specificity. For instance, the genes related to Amino acid metabolism, energy metabolism, Translation, and Metabolism of cofactors and vitamins mainly existed in the rumen, while the gene families associated with Carbohydrate metabolism and Membrane transport mainly consisted in the hindgut. The genes related to Carbohydrate metabolism increased firstly then decreased as goats aged, which matched with several transitional bacteria. This may be ascribed to the switching of diets in the growing process of goats. It is obtained that in the rumen, the microbiota in digesta may contribute more to carbohydrate degradation, while mucosal bacteria mainly participate in energy metabolism. In the cecum, compared with mucosa, the bacterial community in digesta were mainly associated with amino acid metabolism. Nevertheless, a different result was observed in a study on the gastrointestinal microbiota in dairy cattle, which may be due to the difference in species, the stage of growth and development, or diets (Zhang et al., 2021). It is evidenced that breeds, age, diets, or surrounding environment can have a significant influence on the structure of the bacterial community in the GITs, and further affect an animal’s physiological function (Li F. et al., 2019).

In conclusion, the composition of the bacterial community in the rumen differs significantly from in the hindgut and the bacterial community shows spatiotemporal specificity. The similarity of bacterial community within or between each age group increases as goats aged. In the rumen, the composition of the bacterial community in digesta differs significantly from in mucosa. In the hindgut, there is a high similarity of microbiota between in digesta and mucosa in each age group in the pre-weaning period; but after weaning, the distribution of bacterial community is distinct. A total of 25 and 21 core genera were detected in digesta and mucosa of the rumen, cecum and colon, respectively. Whatever in digesta or mucosa, these core genera include the leading, transitional, and mature taxa.

## Data availability statement

The 16S rRNA data of digesta samples (45) before weaning are available from the National Center for Biotechnology Information (NCBI) under accession SRP195450. And the accession number of other 98 samples in NCBI is SRP427554.

## Ethics statement

The animal study was reviewed and approved by the Experimental Animal Management Committee of the Shanxi Agricultural University.

## Author contributions

BL, XW, YY, and YC designed the experiments. BL, WY, and ML performed the experiment. BL carried out microbial data processing and analysis and wrote the manuscript. CZ revised the manuscript and performed the final review, contributed to the project administration, and funding acquisition. All authors contributed to the article and approved the submitted version.

## Footnotes

1
http://www.genome.jp/kegg/
