# Supplementary material for: Exploring the digesta- and mucosa-associated microbial community dynamics in the rumen and hindgut of goats from birth to adult
Source: Front Microbiol. 2023 Jun 15;14:1190348. doi: 10.3389/fmicb.2023.1190348 (PMC10311480; doi:10.3389/fmicb.2023.1190348)
Supplement: Supplementary file 1 [file Data_Sheet_1.docx]

**Exploring the digesta- and mucosa-associated microbial community dynamics in rumen and hindgut of goats from birth to adult**

Bibo Li^1, 2^,Weiqi Yin^1^, Mingkai Lei^1^, Xiaolong Wang^2^, Yuxin Yang^2^, Chunxiang Zhang^1*^ and Yulin Chen^2^*

*^1^College of Animal Science, Shanxi Agriculture University, Taigu, 030801, China*

*^2^Key Laboratory of Animal Genetics, Breeding and Reproduction of Shaanxi Province, College of Animal Science and Technology, Northwest A&F University, Xianyang 712100, China*

**Running Title:** Gut microbiome of goats

***Correspondence:** [chunxiangzhang@sxau.edu.cn](mailto:chunxiangzhang@sxau.edu.cn) (CZ); chenyulin@nwafu.edu.cn (YC)

**Email:**

Bibo Li: [libibo1988@126.com](mailto:libibo1988@126.com)

Weiqi Yin: yin18434762771@163.com

Mingkai Lei: electorboy@163.com

Xiaolong Wang: xiaolongwang[@nwafu.edu.cn](mailto:ｘｉａｏｌｏｎｇｗａｎｇ@nwafu.edu.cn)

Yuxin Yang: [yangyuxin2002@126.com](mailto:yangyuxin2002@126.com)

Chunxiang Zhang: [chunxiangzhang@sxau.edu.cn](mailto:chunxiangzhang@sxau.edu.cn)

Yulin Chen: [chenyulin@nwafu.edu.cn](mailto:chenyulin@nwafu.edu.cn)


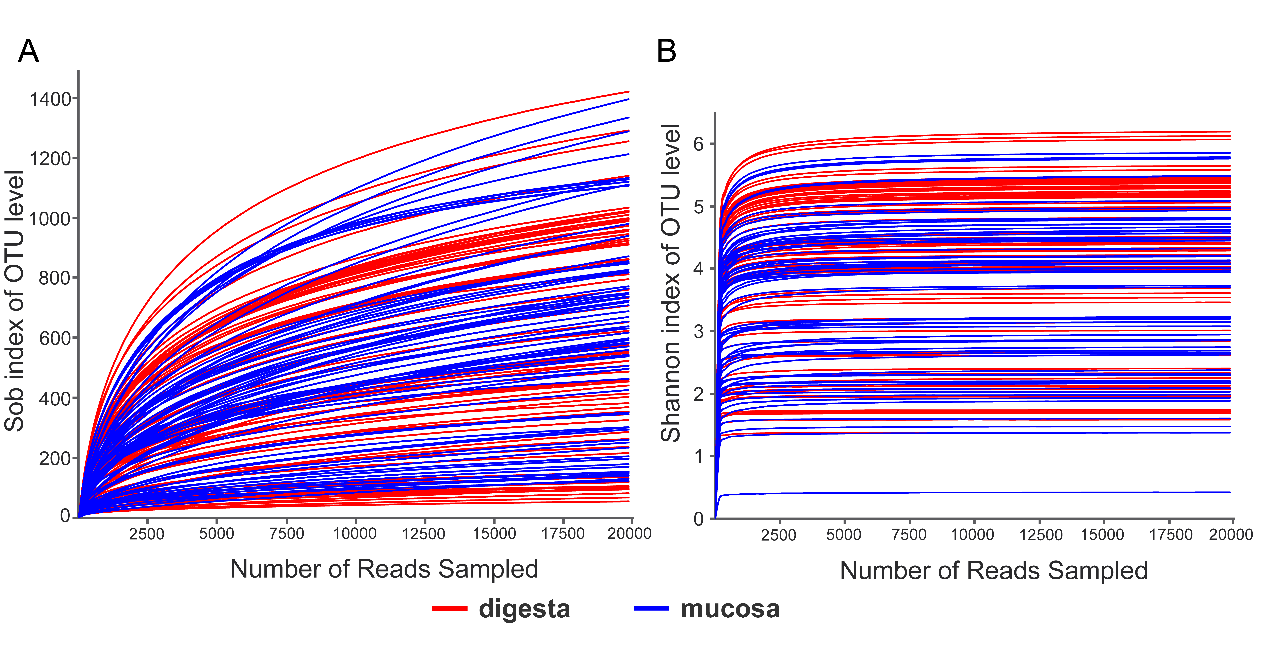
**Fig. S1 Summary of rarefaction results based on operational taxonomic unit (OTUs) (3% divergence) for each sample.**


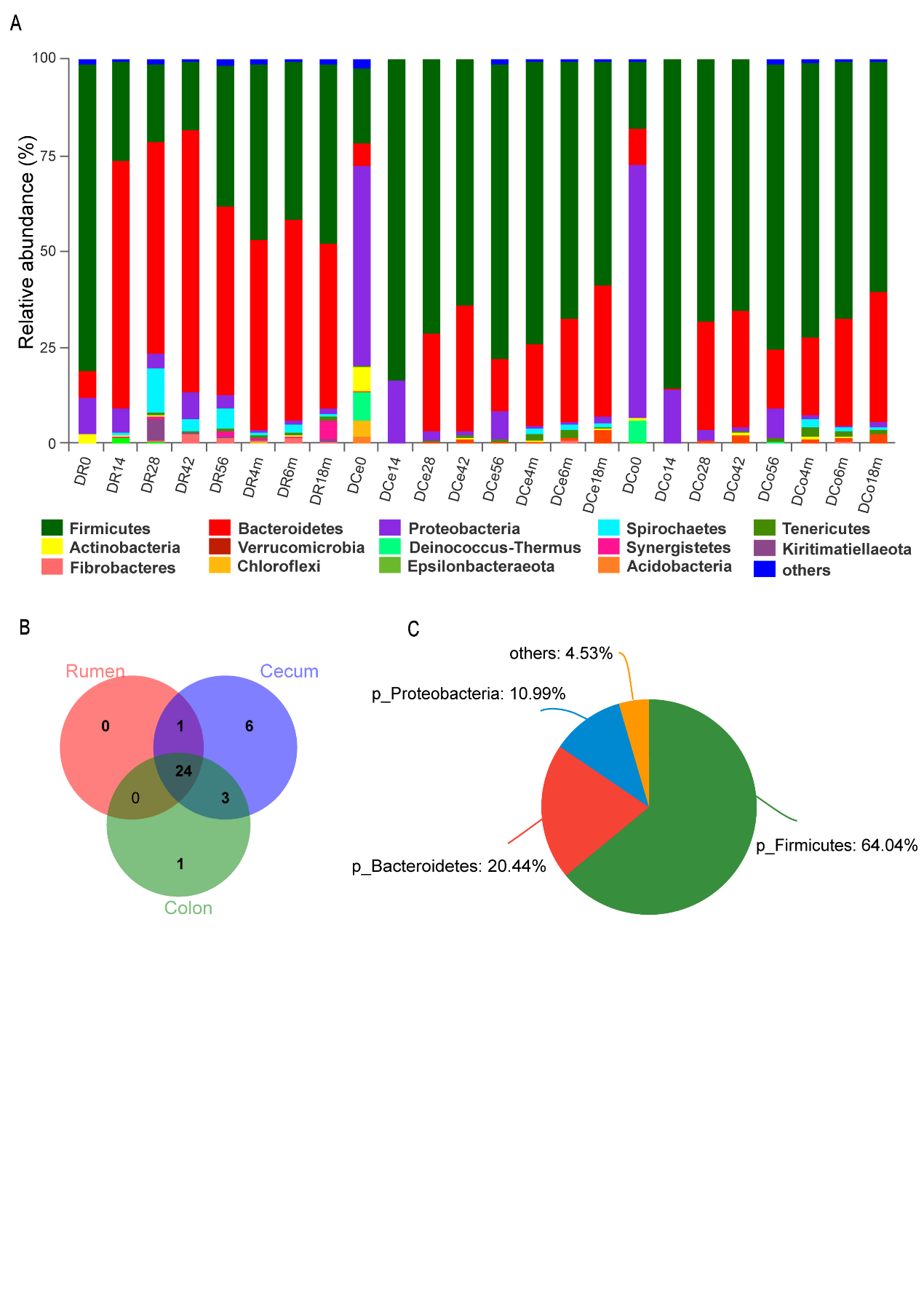


**Fig. S2 Bacterial community composition of digesta (at the phylum level).** (A) Bacterial phyla composition of rumen, cecum and colon at different age. (B) Venn diagram of bacterial phyla shared between rumen, cecum, and colon. (C) Relative proportion of predominant common phyla (those with an average proportion ≥1% based on all common phyla). DR0, DR14, DR28, DR42, DR56, DR4m, DR6m and DR18m represents the rumen digesta sample at 0, 14, 28, 42, 56 days of age, 4, 6 and 18 months of age (or 1.5 years old), respectively. DCe0, DCe14, DCe28, DCe42, DCe56, DCe4m, DCe6m and DCe18m represents cecal digesta sample at 0, 14, 28, 42, 56 days of age, 4, 6 and 18 months of age (or 1.5 years old), respectively. DCo0, DCo14, DCo28, DCo42, DCo56, DCo4m, DCo6m and DCo18m respectively represent colonal digesta sample at 0, 14, 28, 42, 56 days of age, 4, 6 and 18 months of age (or 1.5 years old), respectively.


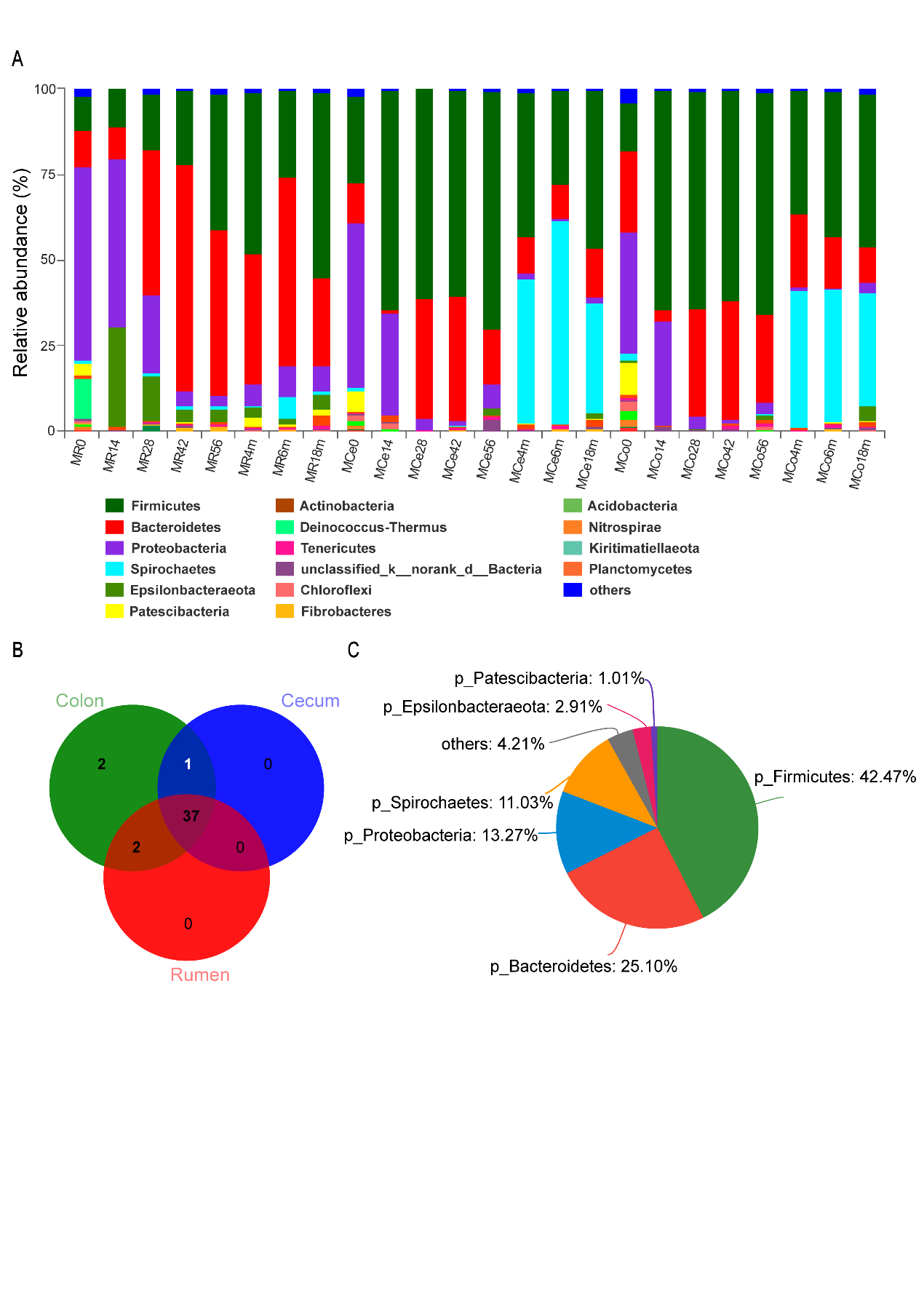


**Fig. S3 Bacterial community composition of mucosal sample at the phylum level.** (A) Bacterial phyla composition of rumen, cecum and colon at different age. (B) Venn diagram of bacterial phyla shared between rumen, cecum, and colon. (C) Relative proportion of predominant common phyla (those with an average proportion ≥1% based on all common phyla). MR0, MR14, MR28, MR42, MR56, MR4m, MR6m and MR18m represent the rumen mucosal sample at 0, 14, 28, 42, 56 days of age, 4, 6 and 18 months of age (or 1.5 years old), respectively. MCe0, MCe14, MCe28, MCe42, MCe56, MCe4m, MCe6m and MCe18m represent cecal mucosa sample at 0, 14, 28, 42, 56 days of age, 4, 6 and 18 months of age (or 1.5 years old), respectively. MCo0, MCo14, MCo28, MCo42, MCo56, MCo4m, MCo6m and MCo18m respectively represent colonal mucosa sample at 0, 14, 28, 42, 56 days of age, 4, 6 and 18 months of age (or 1.5 years old), respectively.


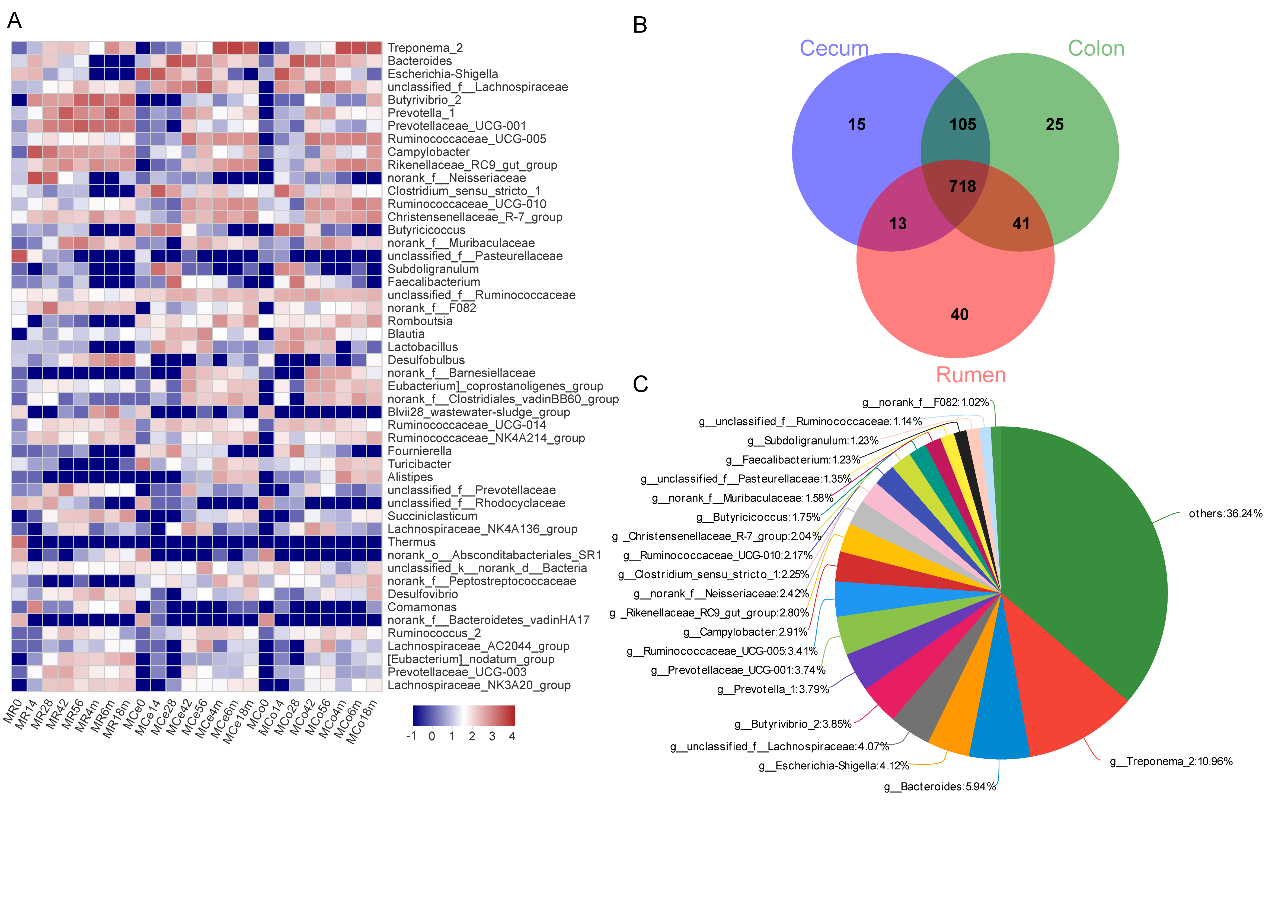


**Fig. S4 Bacterial community composition of mucosa sample at the genus level.** (A) Bacterial phyla composition of rumen, cecum and colon at different ages (the top 50 genera). (B) Venn diagram of bacterial genera shared between rumen, cecum, and colon. (C) Relative proportion of predominant common genera (those with an average proportion ≥1% based on all common genera).


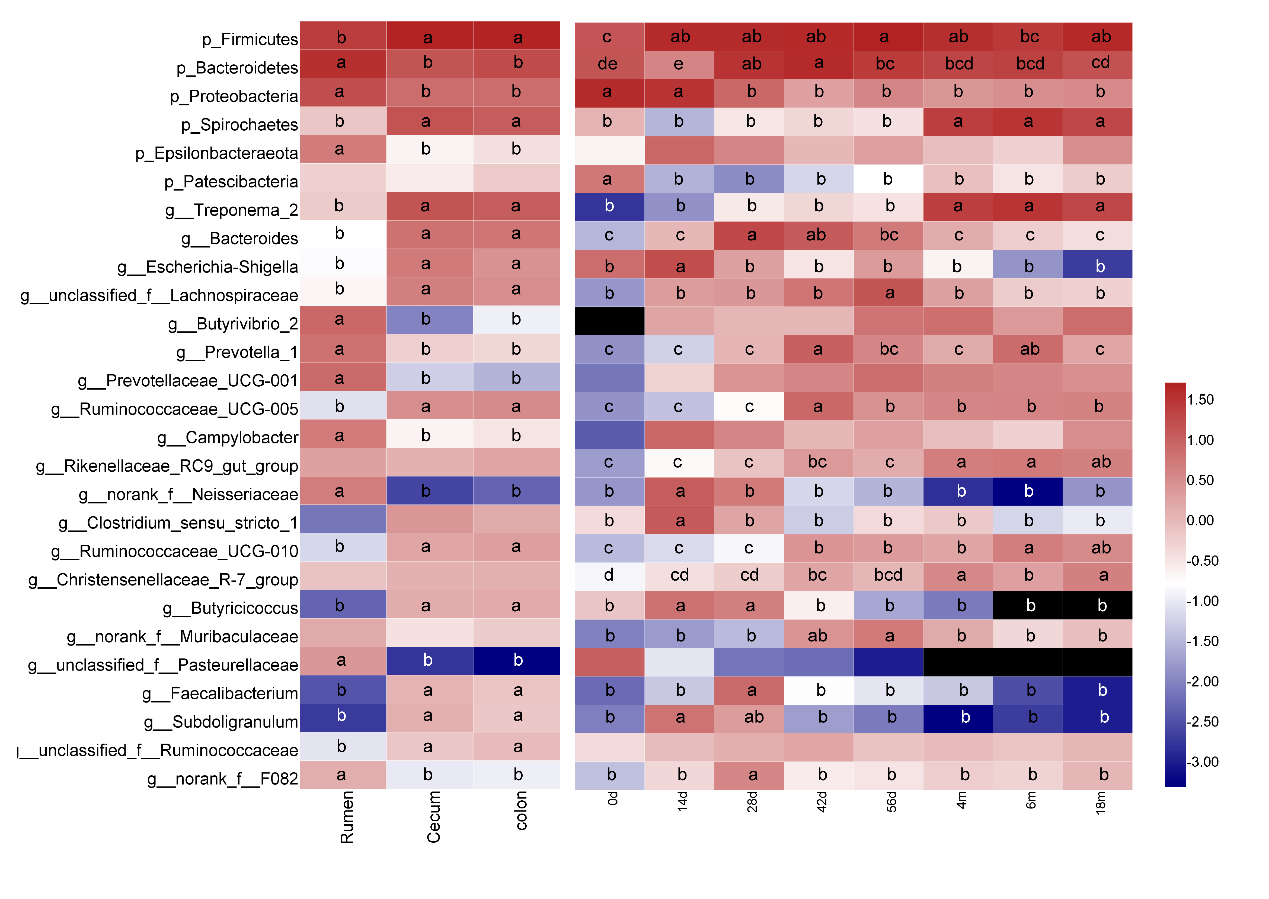


**Fig. S5 Comparison of common dominant bacterial taxa (sequences summarized at phylum [p_] and genus [g_] levels at average abundance of ≥1%) according to GIT region or age group (A).** Means followed by the different letter are significantly different (*P*＜0.05). Taxon rows followed by the same letter or without letters were not significantly different for that variable (*P*＞0.05).


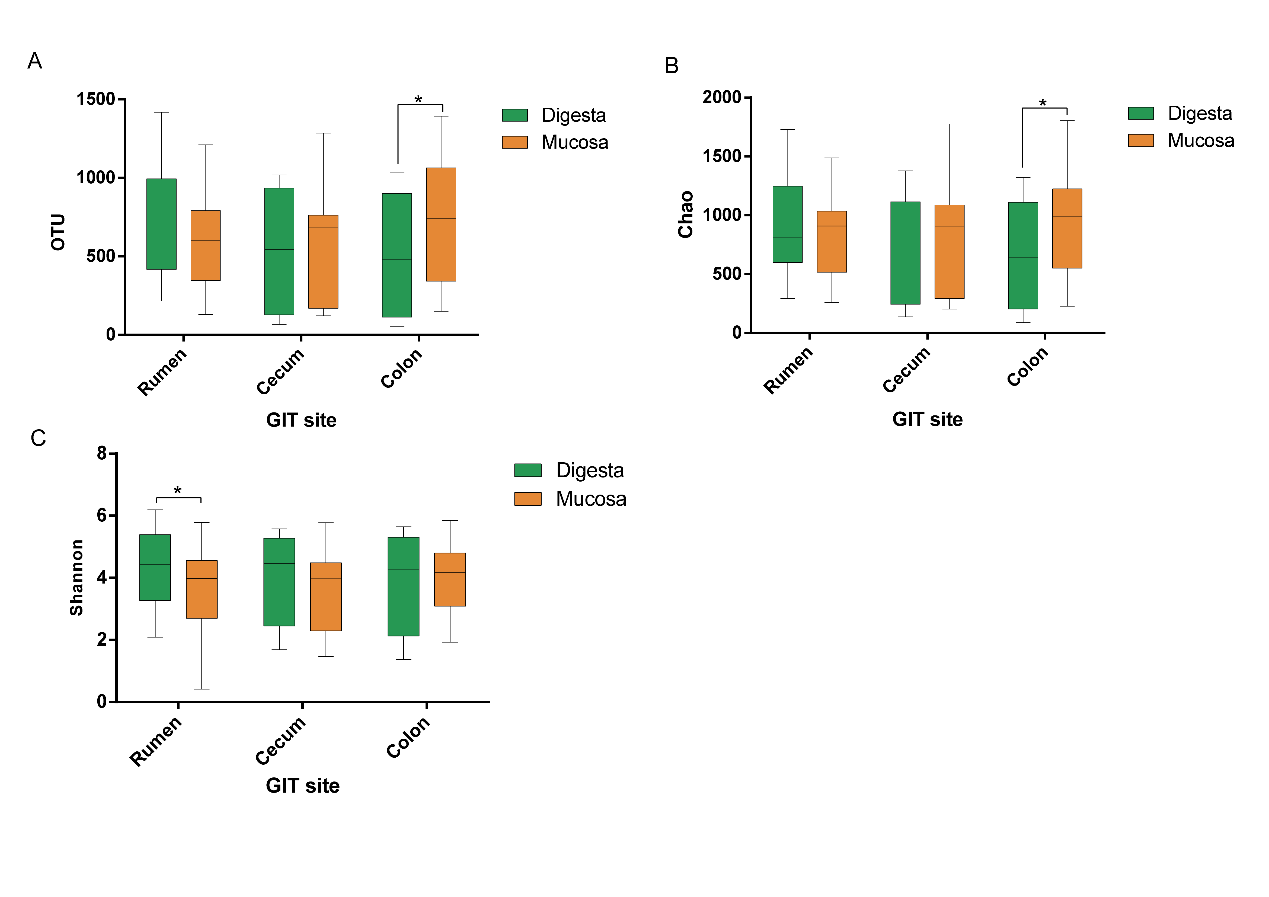


**Fig. S6 Comparison of bacterial community OTU, Chao and Shannon between in digesta and mucosa of rumen, cecum and colon.** Bars with a star symbol above their whiskers are signiﬁcantly different between digesta and mucosa sample using T-test analysis; “*” means “0.01＜*P*＜0.05”, “**” means “0.001＜*P*＜0.01”, “***” means “*P* < 0.001”.


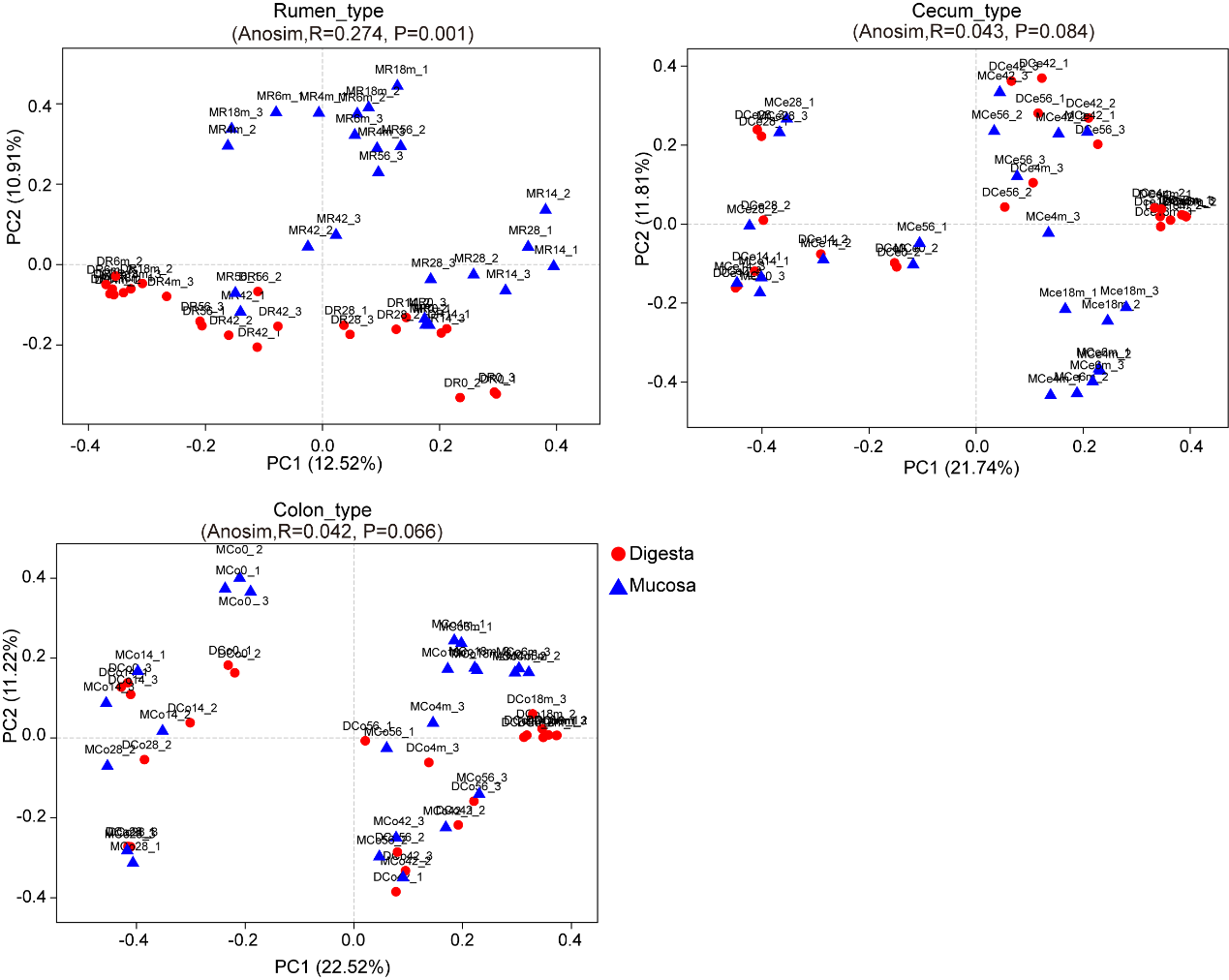


**Fig. S7 Principal coordinate analysis (PCoA) profile of microbial diversity using the Bray-Curtis dissimilarity metric.** The percentage of variation explained by PC1 and PC2 are indicated on the axis.


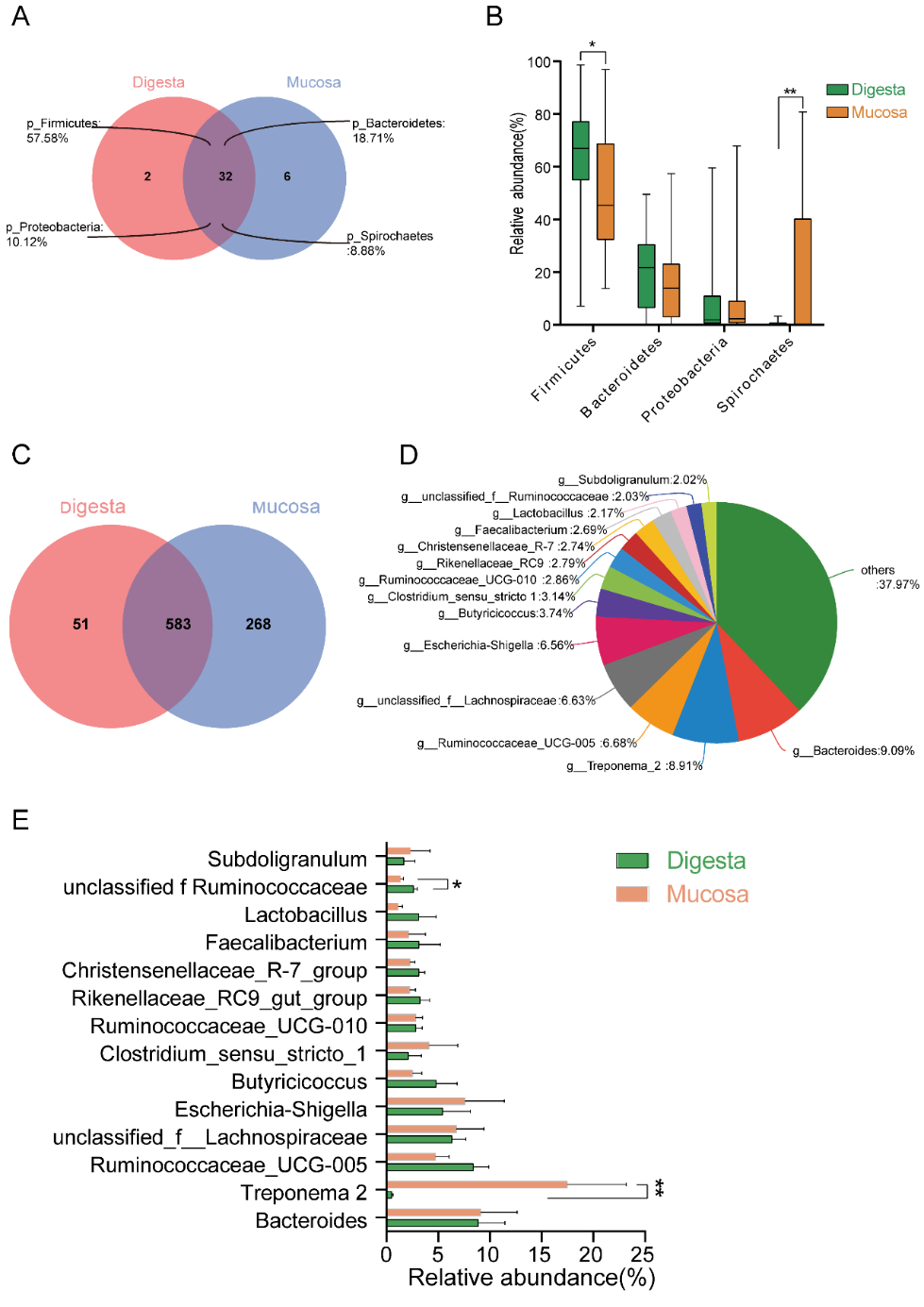
**Fig. S8 Comparison of bacterial taxa between digesta and mucosa sample in cecum.** (A) Venn diagram of bacterial phyla shared between in sample of digesta and mucosa sample. (B) Comparison of common predominant bacterial phyla between digesta and mucosa sample (those with an average proportion ≥1%). (C) Venn diagram of bacterial genera shared between in digesta and mucosa sample. (D) Predominant genera composition shared between digesta and mucosa sample (those with an average proportion ≥2%). (E) Comparison of common predominant bacterial genera between digesta and mucosa sample. Bars with a star symbol above their whiskers are signiﬁcantly different between digesta and mucosa sample using T-test analysis; “*” means “0.01＜*P*＜0.05”, “**” means “0.001＜*P*＜0.01”, “***” means “*P* < 0.001”. The same as below in Fig. S9.


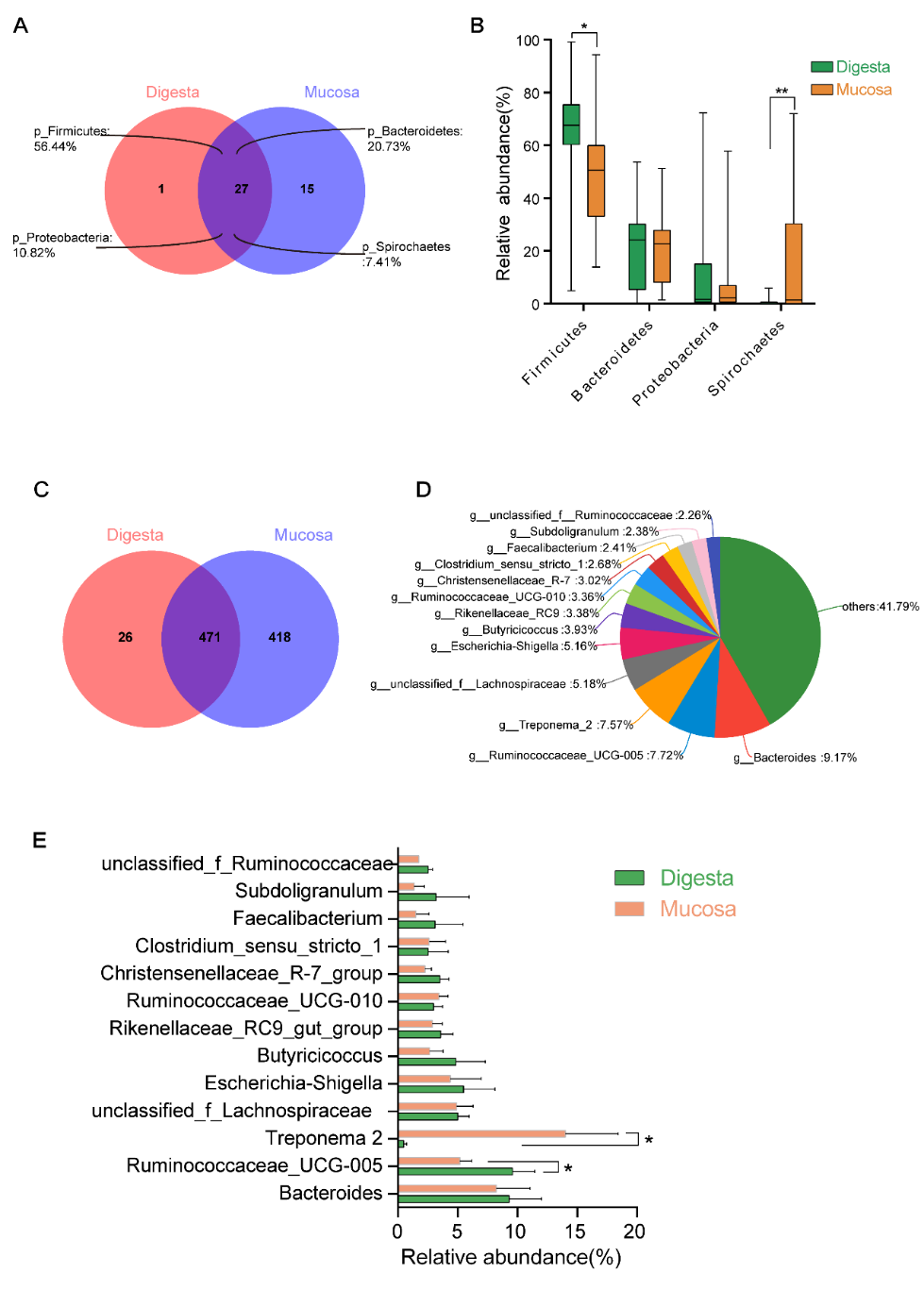


**Fig. S9 Comparison of bacterial taxa between digesta and mucosa sample in colon.** (A) Venn diagram of bacterial phyla shared between in sample of digesta and mucosa sample. (B) Comparison of common predominant bacterial phyla between digesta and mucosa sample (those with an average proportion ≥1%). (C) Venn diagram of bacterial genera shared between in digesta and mucosa sample. (D) Predominant genera composition shared between in digesta and mucosa sample (those with an average proportion ≥2%). (E) Comparison of common predominant bacterial genera between in digesta and mucosa sample.

**Table S1 Ingredients and nutrients of the experimental diets.**

| **Ingredients** | **Content (%)** | **Nutrient level** | **Content** |
| --- | --- | --- | --- |
| Alfalfa meal | 10.00 | Dry matter (DM), % | 91.30 |
| Corn straw | 50.00 | Digestible energy(MJ/kg） | 9.03 |
| Corn | 23.60 | Crude protein, % | 10.38 |
| Wheat bran | 6.00 | crude fat, % | 2.37 |
| Soybean meal | 2.96 | NFE, % | 42.38 |
| Cottonseed meal | 0.56 | ADF, % | 23.13 |
| Rapeseed meal | 5.00 | Calcium, % | 0.47 |
| CaCO_3_ | 0.08 | Phosphorus, % | 0.35 |
| CaHPO_4_ | 0.30 |  |  |
| NaCl | 0.50 |  |  |
| Premix | 1.00 |  |  |
| Total | 100.00 |  |  |

The premix provided the following staff for per kg diet: VA 1 000 IU, VD 200 IU, VE 20 IU,

Fe 40 mg, Zn 30 mg, Cu 15 mg, I 2 mg, Mn 40 mg.

**Table S2 Summary of reads of bacteria from rumen, cecum and colon digesta and mucosa samples of goats according to age group.**

|  |  |  | **Prior to normalization** | | | | | | | | **After to normalization** | | | | | |
| --- | --- | --- | --- | --- | --- | --- | --- | --- | --- | --- | --- | --- | --- | --- | --- | --- |
|  |  |  | **Reads** | | | **OTU** | | | **Coverage** | | **Reads** | **OTU** | | | **Coverage** | |
| **GIT** | **Age** | **Goat/n** | **Total** | **Mean** | **SEM^1^** | **Total** | **Mean** | **SEM** | **Mean** | **SEM** | **Mean** | **Total** | **Mean** | **SEM** | **Mean** | **SEM** |
| **Digesta** |  |  |  |  |  |  |  |  |  |  |  |  |  |  |  |  |
| **Rumen** | 0d | 3 | 108142 | 36047.33 | 1816.559 | 1801.00 | 600.33 | 87.20 | 0.9939 | 0.0009 | 19816 | 1430.00 | 476.67 | 62.56 | 0.9909 | 0.0015 |
|  | 14d | 3 | 95472 | 31824 | 2713.841 | 954.00 | 318.00 | 69.78 | 0.9970 | 0.0006 | 19816 | 818.00 | 272.67 | 48.02 | 0.9955 | 0.0011 |
|  | 28d | 3 | 90840 | 30280 | 1624.762 | 1913.00 | 637.67 | 61.94 | 0.9937 | 0.0005 | 19816 | 1656.00 | 552.00 | 48.04 | 0.9914 | 0.0007 |
|  | 42d | 3 | 83617 | 27872.33 | 2288.526 | 1418.00 | 472.67 | 64.51 | 0.9945 | 0.0007 | 19816 | 1278.00 | 426.00 | 66.34 | 0.9931 | 0.0009 |
|  | 56d | 3 | 80426 | 26808.67 | 717.945 | 2273.00 | 757.67 | 131.80 | 0.9926 | 0.0011 | 19816 | 2116.00 | 705.33 | 123.69 | 0.9896 | 0.0016 |
|  | 4m | 3 | 121051 | 40350.33 | 296.144 | 3453.00 | 1151.00 | 41.10 | 0.9938 | 0.0004 | 19816 | 2926.00 | 975.33 | 28.04 | 0.9870 | 0.0001 |
|  | 6m | 3 | 151336 | 50445.33 | 3510.882 | 3757.00 | 1252.33 | 85.12 | 0.9952 | 0.0004 | 19816 | 3064.00 | 1021.33 | 61.55 | 0.9870 | 0.0002 |
|  | 18m | 3 | 110418 | 36806 | 2296.498 | 4513.00 | 1504.33 | 53.90 | 0.9922 | 0.0007 | 19816 | 3960.00 | 1320.00 | 50.09 | 0.9847 | 0.0005 |
| **Cecum** | 0d | 3 | 98803 | 32934.33 | 2602.43 | 1377.00 | 459.00 | 221.17 | 0.9967 | 0.0009 | 19816 | 1189.00 | 396.33 | 214.06 | 0.9946 | 0.0018 |
|  | 14d | 3 | 115505 | 38501.67 | 3377.05 | 380.00 | 126.67 | 11.67 | 0.9984 | 0.0002 | 19816 | 286.00 | 95.33 | 15.41 | 0.9976 | 0.0004 |
|  | 28d | 3 | 109392 | 36464 | 1558.677 | 473.00 | 157.67 | 19.19 | 0.9984 | 0.0004 | 19816 | 363.00 | 121.00 | 16.52 | 0.9980 | 0.0004 |
|  | 42d | 3 | 94897 | 31632.33 | 3189.559 | 1640.00 | 546.67 | 50.71 | 0.9955 | 0.0005 | 19816 | 1447.00 | 482.33 | 39.01 | 0.9935 | 0.0007 |
|  | 56d | 3 | 106101 | 35367 | 3265.886 | 2319.00 | 773.00 | 64.51 | 0.9938 | 0.0011 | 19816 | 1975.00 | 658.33 | 61.13 | 0.9902 | 0.0010 |
|  | 4m | 3 | 138254 | 46084.67 | 3636.148 | 2813.00 | 937.67 | 202.08 | 0.9954 | 0.0004 | 19816 | 2296.00 | 765.33 | 167.34 | 0.9895 | 0.0015 |
|  | 6m | 3 | 129370 | 43123.33 | 2258.402 | 3452.00 | 1150.67 | 30.56 | 0.9950 | 0.0003 | 19816 | 2940.00 | 980.00 | 18.90 | 0.9883 | 0.0006 |
|  | 18m | 3 | 132781 | 44260.33 | 2193.482 | 3504.00 | 1168.00 | 54.84 | 0.9941 | 0.0010 | 19816 | 2887.00 | 962.33 | 31.80 | 0.9875 | 0.0014 |
| **Colon** | 0d | 3 | 99031 | 33010.33 | 1879.285 | 827.00 | 275.67 | 79.45 | 0.9977 | 0.0004 | 19816 | 708.00 | 236.00 | 71.06 | 0.9964 | 0.0010 |
|  | 14d | 3 | 94831 | 31610.33 | 2536.147 | 293.00 | 97.67 | 14.84 | 0.9986 | 0.0003 | 19816 | 231.00 | 77.00 | 12.29 | 0.9983 | 0.0003 |
|  | 28d | 3 | 95830 | 31943.33 | 1833.437 | 402.00 | 134.00 | 10.50 | 0.9987 | 0.0001 | 19816 | 343.00 | 114.33 | 10.35 | 0.9982 | 0.0002 |
|  | 42d | 3 | 89336 | 29778.67 | 2758.79 | 1509.00 | 503.00 | 50.86 | 0.9959 | 0.0008 | 19816 | 1361.00 | 453.67 | 40.76 | 0.9942 | 0.0011 |
|  | 56d | 3 | 105053 | 35017.67 | 3917.327 | 2218.00 | 739.33 | 83.08 | 0.9944 | 0.0013 | 19816 | 1916.00 | 638.67 | 73.30 | 0.9910 | 0.0011 |
|  | 4m | 3 | 128812 | 42937.33 | 1460.846 | 2739.00 | 913.00 | 185.83 | 0.9952 | 0.0008 | 19816 | 2297.00 | 765.67 | 161.47 | 0.9897 | 0.0019 |
|  | 6m | 3 | 123647 | 41215.67 | 5529.053 | 3389.00 | 1129.67 | 73.33 | 0.9942 | 0.0010 | 19816 | 2913.00 | 971.00 | 56.07 | 0.9875 | 0.0009 |
|  | 18m | 3 | 124662 | 41554 | 374.004 | 3334.00 | 1111.33 | 20.38 | 0.9949 | 0.0002 | 19816 | 2839.00 | 946.33 | 16.90 | 0.9886 | 0.0004 |
| **Mucosa** |  |  |  |  |  |  |  |  |  |  |  |  |  |  |  |  |
| **Rumen** | 0d | 3 | 93516 | 31172 | 6303.16 | 1743.00 | 581.00 | 274.94 | 0.9946 | 0.0019 | 19816 | 708.00 | 530.67 | 300.21 | 0.9938 | 0.0015 |
|  | 14d | 3 | 99252 | 33084 | 1497.016 | 902.00 | 300.67 | 47.61 | 0.9963 | 0.0008 | 19816 | 231.00 | 243.33 | 34.11 | 0.9949 | 0.0009 |
|  | 28d | 3 | 106921 | 35640.33 | 3617.964 | 1949.00 | 649.67 | 160.93 | 0.9936 | 0.0017 | 19816 | 343.00 | 536.00 | 151.92 | 0.9903 | 0.0018 |
|  | 42d | 3 | 93123 | 31041 | 3564.746 | 1850.00 | 616.67 | 64.51 | 0.9933 | 0.0010 | 19816 | 1361.00 | 533.00 | 61.23 | 0.9907 | 0.0011 |
|  | 56d | 3 | 94660 | 31553.33 | 3536.095 | 2106.00 | 702.00 | 138.12 | 0.9928 | 0.0017 | 19816 | 1916.00 | 606.33 | 136.53 | 0.9892 | 0.0025 |
|  | 4m | 3 | 133269 | 44423 | 3271.454 | 2648.00 | 882.67 | 88.56 | 0.9950 | 0.0004 | 19816 | 2297.00 | 714.67 | 76.81 | 0.9889 | 0.0002 |
|  | 6m | 3 | 112095 | 37365 | 844.724 | 2458.00 | 819.33 | 66.91 | 0.9935 | 0.0002 | 19816 | 2913.00 | 677.33 | 74.19 | 0.9881 | 0.0004 |
|  | 18m | 3 | 119521 | 39840.33 | 2035.55 | 3265.00 | 1088.33 | 223.28 | 0.9927 | 0.0009 | 19816 | 2839.00 | 879.33 | 188.27 | 0.9862 | 0.0021 |
| **Cecum** | 0d | 2 | 60030 | 30015 | 9958 | 1296.00 | 648.00 | 458.00 | 0.9948 | 0.0030 | 19816 | 1592.00 | 622.50 | 482.50 | 0.9940 | 0.0025 |
|  | 14d | 3 | 118419 | 39473 | 1888.508 | 1140.00 | 380.00 | 172.03 | 0.9970 | 0.0010 | 19816 | 730.00 | 315.00 | 157.64 | 0.9944 | 0.0019 |
|  | 28d | 3 | 121266 | 40422 | 1453.234 | 569.00 | 189.67 | 13.04 | 0.9980 | 0.0002 | 19816 | 1608.00 | 138.00 | 8.96 | 0.9971 | 0.0002 |
|  | 42d | 3 | 114087 | 38029 | 847.775 | 2206.00 | 735.33 | 95.57 | 0.9947 | 0.0004 | 19816 | 1599.00 | 604.33 | 75.03 | 0.9907 | 0.0009 |
|  | 56d | 3 | 97020 | 32340 | 1384.787 | 2356.00 | 785.33 | 72.14 | 0.9922 | 0.0011 | 19816 | 1819.00 | 662.33 | 60.17 | 0.9886 | 0.0017 |
|  | 4m | 3 | 89666 | 29888.67 | 894.11 | 2201.00 | 733.67 | 65.34 | 0.9919 | 0.0005 | 19816 | 2144.00 | 633.67 | 69.41 | 0.9886 | 0.0008 |
|  | 6m | 3 | 104131 | 34710.33 | 3003.935 | 3130.00 | 1043.33 | 150.84 | 0.9903 | 0.0015 | 19816 | 2032.00 | 868.67 | 120.46 | 0.9835 | 0.0022 |
|  | 18m | 3 | 112121 | 37373.67 | 6549.491 | 3920.00 | 1306.67 | 113.39 | 0.9890 | 0.0014 | 19816 | 2638.00 | 1067.33 | 109.94 | 0.9813 | 0.0016 |
| **Colon** | 0d | 3 | 74938 | 24979.33 | 2175.469 | 3456.00 | 1152.00 | 16.64 | 0.9938 | 0.0007 | 19816 | 3362.00 | 1120.67 | 7.42 | 0.9909 | 0.0003 |
|  | 14d | 3 | 99027 | 33009 | 1820.916 | 963.00 | 321.00 | 43.10 | 0.9966 | 0.0008 | 19816 | 797.00 | 265.67 | 34.84 | 0.9949 | 0.0008 |
|  | 28d | 3 | 109260 | 36420 | 310.82 | 810.00 | 270.00 | 40.73 | 0.9969 | 0.0005 | 19816 | 605.00 | 201.67 | 28.60 | 0.9956 | 0.0008 |
|  | 42d | 3 | 90896 | 30298.67 | 762.566 | 2001.00 | 667.00 | 82.68 | 0.9941 | 0.0010 | 19816 | 1781.00 | 593.67 | 71.88 | 0.9914 | 0.0013 |
|  | 56d | 3 | 93477 | 31159 | 2598.225 | 2939.00 | 979.67 | 280.94 | 0.9907 | 0.0036 | 19816 | 2625.00 | 875.00 | 273.84 | 0.9868 | 0.0048 |
|  | 4m | 3 | 129096 | 43032 | 2087.954 | 2644.00 | 881.33 | 97.84 | 0.9935 | 0.0002 | 19816 | 2001.00 | 667.00 | 77.11 | 0.9882 | 0.0008 |
|  | 6m | 3 | 121950 | 40650 | 3113.495 | 3368.00 | 1122.67 | 149.06 | 0.9925 | 0.0009 | 19816 | 2726.00 | 908.67 | 115.78 | 0.9852 | 0.0027 |
|  | 18m | 3 | 122944 | 40981.33 | 4963.943 | 3892.00 | 1297.33 | 141.49 | 0.9905 | 0.0023 | 19816 | 3124.00 | 1041.33 | 147.34 | 0.9824 | 0.0022 |

^1^Standard error of the mean.

**Table S3 Alpha diversity of bacterial community according to gastrointestinal tract (GIT) region, age and their interaction (GIT*age) in digesta and mucosa sample.**

| **Factor** |  | OTU | Chao | Shannon |
| --- | --- | --- | --- | --- |
| **Digesta** |  |  |  |  |
| **GIT** | Rumen | 718.667^a^ | 932.640^a^ | 4.370^a^ |
|  | Cecum | 557.625^b^ | 726.401^b^ | 4.000^b^ |
|  | Colon | 525.333^b^ | 679.595^b^ | 3.817^b^ |
|  | SEM | 28.697 | 34.145 | 0.111 |
|  | *P* | ＜0.001 | ＜0.001 | 0.003 |
| **Age** | 0d | 369.667^de^ | 514.386^de^ | 2.603^ef^ |
|  | 14d | 148.333^f^ | 245.387^f^ | 2.260^f^ |
|  | 28d | 262.444^ef^ | 378.208^ef^ | 2.887^e^ |
|  | 42d | 454.000^d^ | 595.293^d^ | 4.055^d^ |
|  | 56d | 667.444^c^ | 878.292^c^ | 4.596^c^ |
|  | 4m | 835.444^b^ | 1070.871^b^ | 5.068^bc^ |
|  | 6m | 990.778^a^ | 1227.239 ^ab^ | 5.420^ab^ |
|  | 18m | 1076.222^a^ | 1326.687^a^ | 5.610^a^ |
|  | SEM | 46.862 | 55.759 | 0.180 |
|  | *P* | ＜0.001 | ＜0.001 | ＜0.001 |
| **GIT*Age** | Rumen_0d | 476.667^efg^ | 709.444^def^ | 2.491^fg^ |
|  | Rumen_14d | 272.667^gh^ | 396.464^ghi^ | 3.137^ef^ |
|  | Rumen_28d | 552.000^def^ | 751.508^def^ | 4.088^cd^ |
|  | Rumen_42d | 426.000^fg^ | 577.173^fgh^ | 3.782^de^ |
|  | Rumen_56d | 705.333^cde^ | 913.747^cde^ | 4.585^bcd^ |
|  | Rumen_4m | 975.333^b^ | 1246.359^b^ | 5.363^ab^ |
|  | Rumen_6m | 1021.333^b^ | 1258.812^b^ | 5.388^ab^ |
|  | Rumen_18m | 1320.000^a^ | 1607.613^a^ | 6.124^a^ |
|  | Cecum 0d | 396.333^fg^ | 526.525^fgh^ | 3.089^ef^ |
|  | Cecum 14d | 95.333^h^ | 196.279^i^ | 2.010^g^ |
|  | Cecum 28d | 121.000^h^ | 197.708^i^ | 2.344^fg^ |
|  | Cecum 42d | 482.333^efg^ | 627.122^efg^ | 4.237^cd^ |
|  | Cecum 56d | 658.333^def^ | 893.972^cde^ | 4.608^bcd^ |
|  | Cecum 4m | 765.333^bcd^ | 976.856^bcd^ | 4.895^bc^ |
|  | Cecum 6m | 980.000^b^ | 1186.622^bc^ | 5.497^ab^ |
|  | Cecum 18m | 962.333^b^ | 1206.124^bc^ | 5.317^ab^ |
|  | Colon 0d | 236.000^gh^ | 307.189^hi^ | 2.228^fg^ |
|  | Colon 14d | 77.000^h^ | 143.417^i^ | 1.634^g^ |
|  | Colon 28d | 114.333^h^ | 185.408^i^ | 2.229^fg^ |
|  | Colon 42d | 453.667^efg^ | 581.586^fgh^ | 4.144^cd^ |
|  | Colon 56d | 638.667^def^ | 827.156^def^ | 4.596^bcd^ |
|  | Colon 4m | 765.667^bcd^ | 989.396^bcd^ | 4.945^bc^ |
|  | Colon 6m | 971.000^b^ | 1236.283^b^ | 5.375^ab^ |
|  | Colon 18m | 946.333^bc^ | 1166.325^bc^ | 5.387^ab^ |
|  | SEM | 81.167 | 96.578 | 0.313 |
|  | *P* | ＜0.001 | ＜0.001 | ＜0.001 |
| **Mucosa** |  |  |  |  |
| **GIT** | Rumen | 590.083 | 832.856 | 3.585 |
|  | Cecum | 613.979 | 850.498 | 3.456 |
|  | Colon | 709.208 | 938.548 | 4.049 |
|  | SEM | 48.930 | 57.753 | 0.209 |
|  | *P* | 0.203 | 0.392 | 0.123 |
| **Age** | 0d | 757.944^ab^ | 877.520^bc^ | 4.148^a^ |
|  | 14d | 274.667^c^ | 417.812^d^ | 2.179^c^ |
|  | 28d | 291.889^c^ | 472.014^d^ | 2.866^bc^ |
|  | 42d | 577.000^b^ | 795.995^c^ | 4.354^a^ |
|  | 56d | 714.556^b^ | 978.146^bc^ | 4.038^a^ |
|  | 4m | 671.778^b^ | 934.537^bc^ | 3.691^ab^ |
|  | 6m | 818.222^ab^ | 1150.545^ab^ | 3.762^ab^ |
|  | 18m | 996.000^a^ | 1365.170^a^ | 4.537^a^ |
|  | SEM | 79.903 | 94.311 | 0.341 |
|  | *P* | ＜0.001 | ＜0.001 | ＜0.001 |
| **GIT*Age** | Rumen_0d | 530.667^cdef^ | 681.202^defg^ | 2.961^bcdefg^ |
|  | Rumen_14d | 243.333^def^ | 392.754^efg^ | 1.838^g^ |
|  | Rumen_28d | 536.000^cdef^ | 819.984^cdef^ | 3.516^bcdefg^ |
|  | Rumen_42d | 533.000^cdef^ | 773.418^cdefg^ | 4.194^abcdef^ |
|  | Rumen_56d | 606.333^bcdef^ | 896.672^bcdef^ | 3.509^bcdefg^ |
|  | Rumen_4m | 714.667^abcd^ | 954.310^abcde^ | 4.099^abcdef^ |
|  | Rumen_6m | 677.333^abcde^ | 952.747^abcde^ | 3.763^bcdefg^ |
|  | Rumen_18m | 879.333^abc^ | 1191.759^abcd^ | 4.799^ab^ |
|  | Cecum 0d | 622.500^bcde^ | 719.165^cdefg^ | 3.689^bcdefg^ |
|  | Cecum 14d | 315.000^def^ | 451.755^efg^ | 2.239^fg^ |
|  | Cecum 28d | 138.000^f^ | 246.546^g^ | 2.379^efg^ |
|  | Cecum 42d | 604.333^bcdef^ | 831.146^cdef^ | 4.337^abcde^ |
|  | Cecum 56d | 662.333^abcde^ | 909.402^bcdef^ | 3.781^bcdefg^ |
|  | Cecum 4m | 633.667^bcde^ | 902.726^bcdef^ | 3.449^bcdefg^ |
|  | Cecum 6m | 868.667^abc^ | 1269.254^abc^ | 3.321^bcdefg^ |
|  | Cecum 18m | 1067.333^ab^ | 1473.993^a^ | 4.456^abcd^ |
|  | Colon 0d | 1120.667^a^ | 1232.192^abcd^ | 5.795^a^ |
|  | Colon 14d | 265.667^def^ | 408.927^efg^ | 2.459^defg^ |
|  | Colon 28d | 201.667^ef^ | 349.511^fg^ | 2.704^cdefg^ |
|  | Colon 42d | 593.667^bcdef^ | 783.423^cdefg^ | 4.530^abc^ |
|  | Colon 56d | 875.000^abc^ | 1128.363^abcd^ | 4.825^ab^ |
|  | Colon 4m | 667.000^abcde^ | 946.576^abcde^ | 3.524^bcdefg^ |
|  | Colon 6m | 908.667^abc^ | 1229.635^abcd^ | 4.201^abcdef^ |
|  | Colon 18m | 1041.333^ab^ | 1429.758^ab^ | 4.356^abcde^ |
|  | SEM | 138.396 | 163.351 | 0.590 |
|  | *P* | ＜0.001 | ＜0.001 | 0.003 |

Values refer to mean and standard error of means (SEM); *P*≤ 0.05 were considered significant; Means followed by the same letter are not significantly different (*P* > 0.05) by Tukey HSD test.

**Table S4 Analysis of similarity (ANOSIM)** **of bacterial microbiota according to age in each gastrointestinal tract (GIT) region.** This analysis provides a way to statistically test whether there is a significant difference between two or more groups of samples, by generating a similarity value (R-value) between 0 and 1 using the Bray-Curtis index. R-values closer to 0 represent groups that do not significantly differ, while values closer to 1 represent a highly different community composition. All ANOSIM values were significantly different between the groups at *P* < 0.05.

|  | 0 d | 14 d | 28 d | 42 d | 56 d | 4 m | 6 m | 18 m |  |
| --- | --- | --- | --- | --- | --- | --- | --- | --- | --- |
| **Rumen** |  |  |  |  |  |  |  |  | **Cecum** |
| 0 d |  | 0.185 | 0.444 | 0.482 | 0.407 | 0.778 | 1 | 0.926 | 0 d |
| 14 d | 1 |  | 0.630 | 1 | 0.926 | 1 | 1 | 1 | 14 d |
| 28 d | 1 | 0.667 |  | 0.963 | 1 | 1 | 1 | 1 | 28 d |
| 42d | 1 | 1 | 0.963 |  | 0.370 | 0.444 | 0.889 | 1 | 42d |
| 56 d | 1 | 1 | 0.778 | 0.370 |  | 0.222 | 0.667 | 0.815 | 56 d |
| 4 m | 1 | 1 | 0.778 | 1 | 0.333 |  | 0.185 | 0.370 | 4 m |
| 6 m | 1 | 1 | 0.852 | 0.926 | 0.259 | 0.111 |  | 0.852 | 6 m |
| 18m | 1 | 1 | 0.852 | 1 | 0.407 | 0.740 | 1 |  | 18 m |
| **Colon** |  |  |  |  |  |  |  |  |  |
| 0 d |  |  |  |  |  |  |  |  |  |
| 14 d | 0.593 |  |  |  |  |  |  |  |  |
| 28 d | 0.778 | 0.259 |  |  |  |  |  |  |  |
| 42d | 1 | 1 | 0.852 |  |  |  |  |  |  |
| 56 d | 0.926 | 0.852 | 1 | 0.296 |  |  |  |  |  |
| 4 m | 1 | 1 | 1 | 0.630 | 0.296 |  |  |  |  |
| 6 m | 1 | 1 | 1 | 0.963 | 0.630 | 0.148 |  |  |  |
| 18m | 1 | 1 | 1 | 1 | 0.741 | 0.296 | 0.926 |  |  |

**Table S5 Changes in the relative abundance of bacterial taxa in digesta samples according to gastrointestinal tract region (GIT), age and interaction (GIT*age).**

| **Taxa** | **Region** | | | **SEM** | ***P*** |
| --- | --- | --- | --- | --- | --- |
|  | **Rumen** | **Cecum** | **colon** |  |  |
| p_Firmicutes | 39.341^b^ | 64.327^a^ | 63.732^a^ | 2.467 | ＜0.001 |
| p_Bacteroidetes | 48.761^a^ | 20.105^b^ | 20.776^b^ | 1.945 | ＜0.001 |
| p_Proteobacteria | 4.133^b^ | 10.238^a^ | 11.742^a^ | 1.188 | ＜0.001 |
| p_Spirochaetes | 2.940^a^ | 0.492^b^ | 0.505^b^ | 0.577 | 0.005 |
| *g_**Bacteroides* | 2.016^b^ | 8.850^a^ | 9.313^a^ | 1.782 | 0.009 |
| *g_**Ruminococcaceae UCG-005* | 0.404^b^ | 8.739^a^ | 9.581^a^ | 0.659 | ＜0.001 |
| *g_**Prevotella_1* | 14.456^a^ | 0.130^b^ | 0.071^b^ | 0.693 | ＜0.001 |
| *g_**Rikenellaceae RC9* | 5.659^a^ | 3.246^b^ | 3.583^b^ | 0.568 | 0.008 |
| *g_**unclassified Lachnospiraceae* | 0.880^b^ | 6.330^a^ | 5.002^a^ | 0.777 | ＜0.001 |
| *g_**Escherichia-Shigella* | 0.077^b^ | 5.431^a^ | 5.492^a^ | 2.140 | 0.032 |
| *g_**Butyricicoccus* | 0.005^b^ | 4.807^a^ | 4.868^a^ | 1.376 | 0.022 |
| *g_**Christensenellaceae R-7* | 2.473 | 3.134 | 3.514 | 0.381 | 0.160 |
| *g_**Bacillus* | 6.878^a^ | 0.030^b^ | 0.002 | 0.359b | ＜0.001 |
| *g_**norank F082* | 6.236^a^ | 0.192^b^ | 0.318^b^ | 0.538 | ＜0.001 |
| *g_**Ruminococcaceae UCG-010* | 0.604^b^ | 2.814^a^ | 3.000^a^ | 0.272 | ＜0.001 |
| *g_**Faecalibacterium* | ＜0.001^b^ | 3.147^a^ | 3.114^a^ | 1.465 | 0.029 |
| *g_**unclassified Ruminococcaceae* | 0.820^b^ | 2.614^a^ | 2.548^a^ | 0.291 | ＜0.001 |
| *g_**norank Muribaculaceae* | 3.705^a^ | 0.823^b^ | 1.027^b^ | 0.801 | 0.024 |
| *g_[Eubacterium] coprostanoligenes* | 0.478^b^ | 2.357^a^ | 2.453^a^ | 0.271 | ＜0.001 |
| *g_**Lactobacillus* | 0.211^b^ | 3.109^a^ | 1.720^ab^ | 0.755 | 0.032 |
| *g_**Clostridium_sensu_stricto_1* | 0.029 | 2.123 | 2.533 | 1.143 | 0.261 |
| *g_**Ruminococcaceae NK4A214* | 3.136^a^ | 0.619^b^ | 0.644^b^ | 0.168 | ＜0.001 |
| *g_**Variovorax* | ＜0.001 | 1.528 | 2.441 | 0.846 | 0.031 |
| *g_**Treponema_2* | 2.823^a^ | 0.477^b^ | 0.485^b^ | 0.571 | 0.006 |
| *g_**Stenotrophomonas* | 0.001^b^ | 1.309^a^ | 2.231^a^ | 0.748 | 0.017 |
| *g_Ruminococcaceae UCG-014* | 1.114 | 1.242 | 1.121 | 0.219 | 0.898 |
| *g_**Alistipes* | 0.006^b^ | 1.697^a^ | 1.694^a^ | 0.247 | ＜0.001 |
| *g_**norank_Barnesiellaceae* | 0.009^b^ | 1.636^a^ | 1.483^a^ | 0.411 | 0.013 |
| *g_**Fournierella* | 0.001^b^ | 1.519^a^ | 1.433^a^ | 0.473 | 0.047 |

| **Taxa** | **Age** | | | | | | | | **SEM** | ***P*** |
| --- | --- | --- | --- | --- | --- | --- | --- | --- | --- | --- |
|  | **0d** | **14d** | **28d** | **42d** | **56d** | **4m** | **6m** | **18m** |  |  |
| p_Firmicutes | 39.034^c^ | 65.078^a^ | 53.244^ab^ | 49.025^bc^ | 62.815^a^ | 63.628^a^ | 58.500^ab^ | 55.077^ab^ | 4.028 | ＜0.001 |
| p_Bacteroidetes | 7.533^d^ | 21.657^c^ | 36.405^ab^ | 43.987^a^ | 26.022^c^ | 30.521^bc^ | 35.804^ab^ | 37.114^ab^ | 3.176 | ＜0.001 |
| p_Proteobacteria | 42.500^a^ | 12.191^b^ | 2.904^c^ | 2.974^c^ | 6.119^c^ | 0.848^c^ | 0.615^c^ | 1.485^c^ | 1.940 | ＜0.001 |
| p_Spirochaetes | 0.071 | 0.159 | 3.833 | 0.998 | 1.741 | 1.324 | 1.617 | 0.706 | 0.942 | 0.145 |
| *g_Bacteroides* | 0.116^b^ | 5.103^b^ | 18.157^a^ | 15.787^a^ | 3.985^b^ | 2.869^b^ | 3.874^b^ | 3.924^b^ | 2.910 | ＜0.001 |
| *g_Ruminococcaceae UCG-005* | 0.056^d^ | 0.140^d^ | 0.296^d^ | 11.012^ab^ | 5.952^c^ | 8.923^bc^ | 12.589^a^ | 10.004^ab^ | 1.076 | ＜0.001 |
| *g_Prevotella_1* | 0.498^d^ | 0.275^d^ | 1.794^d^ | 13.447^a^ | 6.572^bc^ | 5.746^bc^ | 7.163^b^ | 3.589^cd^ | 1.131 | ＜0.001 |
| *g_Rikenellaceae RC9* | 0.043^c^ | 0.385^c^ | 1.236^c^ | 0.907^c^ | 2.704^c^ | 7.280^b^ | 10.732^a^ | 10.014^a^ | 0.927 | ＜0.001 |
| *g_unclassified Lachnospiraceae* | 0.153^c^ | 3.038^bc^ | 1.234^c^ | 6.496^ab^ | 9.416^a^ | 6.533^ab^ | 3.584^bc^ | 2.111^c^ | 1.269 | ＜0.001 |
| *g_Escherichia-Shigella* | 13.275^a^ | 9.821^ab^ | 1.434^b^ | 0.308^b^ | 4.345^ab^ | 0.127^b^ | 0.025^b^ | ＜0.001^b^ | 3.495 | 0.043 |
| *g_Butyricicoccus* | 1.788^b^ | 16.606^a^ | 7.103^b^ | 0.237^b^ | 0.026^b^ | 0.053^b^ | ＜0.001^b^ | 0.001^b^ | 2.246 | ＜0.001 |
| *g_Christensenellaceae R-7* | 0.061^c^ | 0.776^c^ | 1.068^c^ | 3.055^b^ | 4.295^ab^ | 5.743^a^ | 5.106^a^ | 4.216^ab^ | 0.622 | ＜0.001 |
| *g_Bacillus* | 18.387^a^ | 0.018^b^ | 0.009^b^ | 0.004^b^ | 0.007^b^ | 0.003^b^ | ＜0.001^b^ | 0.001 | 0.586 | ＜0.001 |
| *g_norank F082* | 0.018^c^ | 5.098^ab^ | 6.423^a^ | 0.107^c^ | 0.721^c^ | 1.243^c^ | 1.554^c^ | 2.827^bc^ | 0.879 | ＜0.001 |
| *g_Ruminococcaceae UCG-010* | 0.007^c^ | 0.170^c^ | 0.164^c^ | 1.706^b^ | 1.704^b^ | 2.097^b^ | 5.879^a^ | 5.386^a^ | 0.444 | ＜0.001 |
| *g_Faecalibacterium* | 0.034^b^ | 0.001^b^ | 15.824^a^ | 0.553^b^ | 0.121^b^ | 0.143^b^ | 0.012^b^ | 0.011^b^ | 2.392 | ＜0.001 |
| *g_unclassified Ruminococcaceae* | 0.847 | 2.005 | 1.667 | 2.193 | 1.468 | 2.789 | 1.992 | 2.992 | 0.476 | 0.058 |
| *g_norank Muribaculaceae* | 0.035^b^ | 0.017^b^ | 0.057^b^ | 1.331^b^ | 6.172^a^ | 3.858^ab^ | 1.616^b^ | 1.729^b^ | 1.309 | 0.018 |
| *g_[Eubacterium] coprostanoligenes* | 0.019^b^ | 0.457^b^ | 0.128^b^ | 2.699^a^ | 1.126^b^ | 2.420^a^ | 3.494^a^ | 3.760^a^ | 0.442 | ＜0.001 |
| *g_Lactobacillus* | 0.631^b^ | 10.882^a^ | 1.154^b^ | 0.167^b^ | 0.605^b^ | 0.001^b^ | ＜0.001^b^ | ＜0.001^b^ | 1.233 | ＜0.001 |
| *g_Clostridium_sensu_stricto_1* | 1.225^b^ | 9.145^a^ | 0.856^b^ | 0.053^b^ | 0.842^b^ | 0.319^b^ | 0.035^b^ | 0.021^b^ | 1.866 | 0.017 |
| *g_Ruminococcaceae NK4A214* | 0.020^d^ | 2.601^a^ | 1.105^bc^ | 0.479^cd^ | 1.111^bc^ | 2.224^a^ | 2.274^a^ | 1.915^ab^ | 0.275 | ＜0.001 |
| *g_Variovorax* | 10.027^a^ | 0.158^b^ | 0.118^b^ | 0.119^b^ | 0.164^b^ | ＜0.001^b^ | ＜0.001^b^ | ＜0.001^b^ | 1.382 | ＜0.001 |
| *g_Treponema_2* | 0.008 | 0.082 | 3.747 | 0.988 | 1.715 | 1.295 | 1.568 | 0.690 | 0.933 | 0.154 |
| *g_Stenotrophomonas* | 8.937^a^ | 0.188^b^ | 0.100^b^ | 0.087^b^ | 0.131^b^ | ＜0.001^b^ | 0.001^b^ | ＜0.001^b^ | 1.222 | ＜0.001 |
| *g_Ruminococcaceae UCG-014* | 0.024^d^ | 0.911^bcd^ | 1.174^bc^ | 0.546^cd^ | 1.201^bc^ | 2.813^a^ | 1.715^b^ | 0.890^bcd^ | 0.357 | ＜0.001 |
| *g_Alistipes* | 0.002^c^ | 0.014^c^ | 0.002^c^ | 0.016^c^ | 0.123^c^ | 3.714^a^ | 1.926^b^ | 3.262^a^ | 0.403 | ＜0.001 |
| *g_norank_Barnesiellaceae* | 0.026^b^ | ＜0.001^b^ | 0.001^b^ | 4.638^a^ | 1.425^b^ | 0.878^b^ | 0.924^b^ | 0.448^b^ | 0.672 | ＜0.001 |
| *g_Fournierella* | 0.472^b^ | 0.885^b^ | 5.624^a^ | 0.127^b^ | 0.118^b^ | 0.643^b^ | 0.007^b^ | ＜0.001^b^ | 0.772 | ＜0.001 |
|  | **Region*Age** | | | | | | | | **SEM** | ***P*** |
| **Taxa** | **Rumen_0d** | **Rumen_14d** | **Rumen_28d** | **Rumen_42d** | **Rumen_56d** | **Rumen_4m** | **Rumen_6m** | **Rumen_18m** |  |  |
| p_Firmicutes | 80.042^abc^ | 25.920^ghi^ | 20.161^hi^ | 17.907^i^ | 36.898^fghi^ | 45.815^defg^ | 41.019^efgh^ | 46.966^defg^ | 6.977 | ＜0.001 |
| p_Bacteroidetes | 6.971^jk^ | 64.576^ab^ | 55.323^abc^ | 68.605^a^ | 49.326^bcd^ | 49.617^bcd^ | 52.603^abc^ | 43.063^cde^ | 5.500 | ＜0.001 |
| p_Proteobacteria | 9.651^cde^ | 6.401^cde^ | 3.627^de^ | 6.976^cde^ | 3.465^de^ | 0.779^e^ | 0.905^e^ | 1.262^e^ | 3.361 | ＜0.001 |
| p_Spirochaetes | 0.024^b^ | 0.476^b^ | 11.648^a^ | 2.991^b^ | 5.193^b^ | 0.505^b^ | 2.110^b^ | 0.572^b^ | 1.631 | 0.018 |
| *g_Bacteroides* | 0.217^b^ | 15.167^ab^ | 0.735^b^ | 0.005^b^ | ＜0.001^b^ | 0.003^b^ | 0.002^b^ | ＜0.001^b^ | 1.367 | 0.019 |
| *g_Ruminococcaceae UCG-005* | 0.098^e^ | 0.417^e^ | 0.760^e^ | 0.019^e^ | 0.271^e^ | 0.740^e^ | 0.246^e^ | 0.685^e^ | 0.928 | ＜0.001 |
| *g_Prevotella_1* | 1.465^d^ | 0.816^d^ | 5.368^cd^ | 40.310^a^ | 18.414^b^ | 17.068^b^ | 21.458^b^ | 10.746^c^ | 1.205 | ＜0.001 |
| *g_Rikenellaceae RC9* | 0.076^d^ | 1.156^d^ | 3.703^d^ | 1.689^d^ | 4.788^cd^ | 14.191^a^ | 10.739^ab^ | 8.934^bc^ | 0.611 | 0.057 |
| *g_unclassified Lachnospiraceae* | 0.301 | 0.126 | 0.478 | 0.274 | 3.190 | 0.713 | 0.797 | 1.159 | 0.619 | 0.283 |
| *g_Escherichia-Shigella* | 0.040 | 0.577 | ＜0.001 | ＜0.001 | ＜0.001 | ＜0.001 | ＜0.001 | ＜0.001 | 1.269 | 0.903 |
| *g_Butyricicoccus* | 0.000 | 0.007 | ＜0.001 | 0.030 | ＜0.001 | ＜0.001 | ＜0.001 | ＜0.001 | 1.076 | 0.065 |
| *g_Christensenellaceae R-7* | 0.143^e^ | 2.291^bcde^ | 3.147^bcde^ | 0.510^de^ | 1.003^cde^ | 4.300^abc^ | 4.529^abc^ | 3.862^abcd^ | 0.340 | 0.013 |
| *g_Bacillus* | 54.915^a^ | 0.054^b^ | 0.027^b^ | 0.007^b^ | 0.019^b^ | 0.003^b^ | ＜0.001^b^ | ＜0.001^b^ | 1.313 | ＜0.001 |
| *g_norank F082* | 0.029^c^ | 15.293^a^ | 19.268^a^ | 0.320^c^ | 2.138^bc^ | 3.595^bc^ | 3.393^bc^ | 5.854^b^ | 0.624 | ＜0.001 |
| *g_Ruminococcaceae UCG-010* | 0.015^c^ | 0.510^bc^ | 0.490^bc^ | 0.040^c^ | 0.150^c^ | 1.048^bc^ | 0.873^bc^ | 1.706^bc^ | 0.351 | ＜0.001 |
| *g_Faecalibacterium* | 0.003^b^ | ＜0.001^b^ | ＜0.001^b^ | ＜0.001^b^ | ＜0.001^b^ | ＜0.001^b^ | ＜0.001^b^ | ＜0.001^b^ | 1.041 | 0.016 |
| *g_unclassified Ruminococcaceae* | 0.044^d^ | 3.997^a^ | 0.747^bcd^ | 0.032^d^ | 0.584^cd^ | 0.328^cd^ | 0.315^cd^ | 0.515^cd^ | 0.220 | 0.011 |
| *g_norank Muribaculaceae* | 0.082 | 0.047 | 0.170 | 3.400 | 11.803 | 6.167 | 3.662 | 4.308 | 0.499 | 0.906 |
| *g_[Eubacterium] coprostanoligenes* | 0.034^d^ | 0.232^d^ | 0.377^d^ | 0.205^d^ | 0.234^d^ | 0.749d | 0.728d | 1.263^cd^ | 0.257 | 0.043 |
| *g_Lactobacillus* | 0.044^c^ | 1.640^c^ | ＜0.001^c^ | ＜0.001^c^ | ＜0.001^c^ | 0.002^c^ | ＜0.001^c^ | ＜0.001^c^ | 0.644 | 0.009 |
| *g_Clostridium_sensu_stricto_1* | 0.204 | 0.030 | 0.002 | ＜0.001 | ＜0.001 | ＜0.001 | ＜0.001 | ＜0.001 | 0.700 | 0.751 |
| *g_Ruminococcaceae NK4A214* | 0.034^d^ | 7.769^a^ | 3.295^c^ | 0.355^d^ | 0.779^d^ | 4.271^bc^ | 4.776^b^ | 3.810^bc^ | 0.239 | ＜0.001 |
| *g_Variovorax* | 0.002^c^ | ＜0.001^c^ | ＜0.001^c^ | ＜0.001^c^ | ＜0.001^c^ | ＜0.001^c^ | ＜0.001^c^ | ＜0.001^c^ | 0.647 | 0.047 |
| *g_Treponema_2* | 0.022^b^ | 0.247^b^ | 11.241^a^ | 2.962^b^ | 5.117^b^ | 0.434^b^ | 2.037^b^ | 0.525^b^ | 0.394 | 0.022 |
| *g_Stenotrophomonas* | 0.008^c^ | ＜0.001^c^ | ＜0.001^c^ | ＜0.001^c^ | ＜0.001^c^ | ＜0.001^c^ | ＜0.001^c^ | ＜0.001^c^ | 0.578 | 0.033 |
| *g_Ruminococcaceae UCG-014* | 0.049 | 2.014 | 1.576 | 0.187 | 0.797 | 1.866 | 1.329 | 1.097 | 0.148 | 0.665 |
| *g_Alistipes* | 0.005^d^ | 0.042^d^ | 0.003^d^ | ＜0.001^d^ | ＜0.001^d^ | ＜0.001^d^ | ＜0.001^d^ | ＜0.001^d^ | 0.267 | ＜0.001 |
| *g_norank_Barnesiellaceae* | 0.067 | ＜0.001 | 0.002 | ＜0.001 | ＜0.001 | 0.002 | ＜0.001 | ＜0.001 | 0.300 | 0.213 |
| *g_Fournierella* | ＜0.001 | 0.007 | ＜0.001 | ＜0.001 | ＜0.001 | ＜0.001 | ＜0.001 | ＜0.001 | 0.353 | 0.125 |
|  | **Cecum_0d** | **Cecum_14d** | **Cecum_28d** | **Cecum_42d** | **Cecum_56d** | **Cecum_4m** | **Cecum_6m** | **Cecum_18m** |  |  |
| p_Firmicutes | 19.687^hi^ | 83.499^ab^ | 71.429^abc^ | 63.997^abcd^ | 77.154^abc^ | 73.458^abc^ | 67.131^abcd^ | 58.264^cdef^ |  |  |
| p_Bacteroidetes | 6.049^jk^ | 0.173^k^ | 25.393^efghi^ | 32.704^defg^ | 13.527^hijk^ | 21.408^fghij^ | 27.166^efghi^ | 34.417^def^ |  |  |
| p_Proteobacteria | 51.871^b^ | 16.249^c^ | 2.574^e^ | 1.097^e^ | 7.160^cde^ | 0.767^e^ | 0.493^e^ | 1.691^e^ |  |  |
| p_Spirochaetes | 0.061^b^ | < 0.001^b^ | < 0.001^b^ | < 0.001^b^ | 0.010^b^ | 1.334^b^ | 1.583^b^ | 0.950^b^ |  |  |
| *g_Bacteroides* | 0.071^b^ | 0.037^b^ | 25.291^a^ | 24.285^a^ | 4.998^b^ | 4.293^b^ | 6.140^b^ | 5.689^b^ |  |  |
| *g_Ruminococcaceae UCG-005* | 0.032^e^ | 0.002^e^ | 0.082^e^ | 13.258^bcd^ | 9.049^cd^ | 12.803^bcd^ | 17.460^ab^ | 14.348^abc^ |  |  |
| *g_Prevotella_1* | 0.020^d^ | 0.007^d^ | 0.013^d^ | 0.029^d^ | 0.888^d^ | 0.047^d^ | 0.013^d^ | 0.020^d^ |  |  |
| *g_Rikenellaceae RC9* | 0.037^d^ | ＜0.001^d^ | 0.002^d^ | 0.427^d^ | 1.810^d^ | 3.834^d^ | 9.449^abc^ | 10.408^ab^ |  |  |
| *g_unclassified Lachnospiraceae* | 0.071 | 7.017 | 1.142 | 10.494 | 12.898 | 10.773 | 5.506 | 2.737 |  |  |
| *g_Escherichia-Shigella* | 18.940 | 15.450 | 2.148 | 0.474 | 6.231 | 0.187 | 0.022 | ＜0.001 |  |  |
| *g_Butyricicoccus* | 3.524 | 22.676 | 11.663 | 0.439 | 0.032 | 0.123 | ＜0.001 | 0.002 |  |  |
| *g_Christensenellaceae R-7* | 0.024^e^ | 0.020^e^ | 0.037^e^ | 4.296^abc^ | 4.857^ab^ | 5.726^ab^ | 5.370^ab^ | 4.739^ab^ |  |  |
| *g_Bacillus* | 0.232^b^ | ＜0.001^b^ | ＜0.001^b^ | 0.005^b^ | 0.002^b^ | 0.005^b^ | ＜0.001^b^ | ＜0.001^b^ |  |  |
| *g_norank F082* | 0.024^c^ | ＜0.001^c^ | ＜0.001^c^ | ＜0.001^c^ | 0.008^c^ | 0.084^c^ | 0.607^c^ | 0.813^c^ |  |  |
| *g_Ruminococcaceae UCG-010* | 0.005^c^ | ＜0.001^c^ | 0.003^c^ | 2.656^bc^ | 2.495^bc^ | 2.949^b^ | 7.942^a^ | 6.458^a^ |  |  |
| *g_Faecalibacterium* | 0.061^b^ | ＜0.001^b^ | 23.745^a^ | 0.920^b^ | 0.182^b^ | 0.237^b^ | 0.019^b^ | 0.013^b^ |  |  |
| *g_unclassified Ruminococcaceae* | 0.913^bcd^ | 1.105^bcd^ | 2.535^abcd^ | 3.519^ab^ | 1.919^abcd^ | 3.948^a^ | 2.890^abc^ | 4.079^a^ |  |  |
| *g_norank Muribaculaceae* | 0.015 | 0.002 | ＜0.001 | 0.296 | 3.129 | 2.096 | 0.612 | 0.437 |  |  |
| *g_[Eubacterium] coprostanoligenes* | 0.013^d^ | 0.547^d^ | 0.002^d^ | 3.857^ab^ | 1.395^cd^ | 3.227^abc^ | 5.030^a^ | 4.788^a^ |  |  |
| *g_Lactobacillus* | 1.792^c^ | 20.971^a^ | 1.216^c^ | 0.286^c^ | 0.607^c^ | ＜0.001^c^ | ＜0.001^c^ | ＜0.001^c^ |  |  |
| *g_Clostridium_sensu_stricto_1* | 0.858 | 12.511 | 1.502 | 0.133 | 1.359 | 0.528 | 0.056 | 0.039 |  |  |
| *g_Ruminococcaceae NK4A214* | 0.025^d^ | 0.030^d^ | 0.008^d^ | 0.503^d^ | 1.393^d^ | 1.122^d^ | 0.885^d^ | 0.982^d^ |  |  |
| *g_Variovorax* | 11.281^b^ | 0.298^c^ | 0.183^c^ | 0.212^c^ | 0.249^c^ | ＜0.001^c^ | ＜0.001^c^ | 0.183^c^ |  |  |
| *g_Treponema_2* | 0.003^b^ | ＜0.001^b^ | ＜0.001^b^ | ＜0.001^b^ | 0.010^b^ | 1.327^b^ | 1.526^b^ | 0.950^b^ |  |  |
| *g_Stenotrophomonas* | 9.599^b^ | 0.331^c^ | 0.172^c^ | 0.170^c^ | 0.200^c^ | ＜0.001^c^ | ＜0.001^c^ | ＜0.001^c^ |  |  |
| *g_Ruminococcaceae UCG-014* | 0.007 | 0.506 | 0.959 | 0.723 | 1.514 | 3.514 | 2.041 | 0.675 |  |  |
| *g_Alistipes* | ＜0.001^d^ | ＜0.001^d^ | ＜0.001^d^ | 0.024^d^ | 0.209^d^ | 6.090^a^ | 3.178^bc^ | 4.076^abc^ |  |  |
| *g_norank_Barnesiellaceae* | 0.007 | ＜0.001 | ＜0.001 | 7.400 | 2.073 | 1.502 | 1.492 | 0.611 |  |  |
| *g_Fournierella* | 0.903 | 0.855 | 9.020 | 0.193 | 0.160 | 1.009 | 0.015 | ＜0.001 |  |  |
|  | **Colon_0d** | **Colon_14d** | **Colon_28d** | **Colon_42d** | **Colon_56d** | **Colon_4m** | **Colon_6m** | **Colon_18m** |  |  |
| p_Firmicutes | 17.374^i^ | 85.814^a^ | 68.140a^bcd^ | 65.171^abcd^ | 74.395^abc^ | 71.611^abc^ | 67.350^abcd^ | 60.000^bcde^ |  |  |
| p_Bacteroidetes | 9.579^ijk^ | 0.222^k^ | 28.499^efgh^ | 30.652^efgh^ | 15.212^ghijk^ | 20.537^fghij^ | 27.642^efghi^ | 33.862^def^ |  |  |
| p_Proteobacteria | 65.977^a^ | 13.924^cd^ | 2.510^e^ | 0.848^e^ | 7.733^cde^ | 0.998^e^ | 0.446^e^ | 1.504^e^ |  |  |
| p_Spirochaetes | 0.130^b^ | < 0.001^b^ | 0.002^b^ | 0.002^b^ | 0.019^b^ | 2.133^b^ | 1.157^b^ | 0.596^b^ |  |  |
| *g_Bacteroides* | 0.059^b^ | 0.104^b^ | 28.445^a^ | 23.070^a^ | 6.958^b^ | 4.310^b^ | 5.479^b^ | 6.081^b^ |  |  |
| *g_Ruminococcaceae UCG-005* | 0.037^e^ | ＜0.001^e^ | 0.045^e^ | 19.759^a^ | 8.537^d^ | 13.226^bcd^ | 20.062^a^ | 14.979^abc^ |  |  |
| *g_Prevotella_1* | 0.008^d^ | 0.003^d^ | 0.002^d^ | 0.002^d^ | 0.416^d^ | 0.123^d^ | 0.017^d^ | ＜0.001^d^ |  |  |
| *g_Rikenellaceae RC9* | 0.017^d^ | ＜0.001^d^ | 0.003^d^ | 0.606^d^ | 1.514^d^ | 3.814^d^ | 12.008^ab^ | 10.701^ab^ |  |  |
| *g_unclassified Lachnospiraceae* | 0.086 | 1.970 | 2.083 | 8.719 | 12.161 | 8.113 | 4.448 | 2.438 |  |  |
| *g_Escherichia-Shigella* | 20.884 | 13.438 | 2.155 | 0.449 | 6.805 | 0.195 | 0.052 | ＜0.001 |  |  |
| *g_Butyricicoccus* | 1.840 | 27.134 | 9.646 | 0.242 | 0.045 | 0.037 | ＜0.001 | 0.002 |  |  |
| *g_Christensenellaceae R-7* | 0.017^e^ | 0.017^e^ | 0.020^e^ | 4.360^abc^ | 7.025^a^ | 7.202^a^ | 5.420^ab^ | 4.047^abc^ |  |  |
| *g_Bacillus* | 0.015^b^ | ＜0.001^b^ | ＜0.001^b^ | ＜0.001^b^ | ＜0.001^b^ | 0.002^b^ | ＜0.001^b^ | ＜0.001^b^ |  |  |
| *g_norank F082* | 0.002^c^ | ＜0.001^c^ | ＜0.001^c^ | ＜0.001^c^ | 0.017^c^ | 0.050^c^ | 0.663^c^ | 1.815^bc^ |  |  |
| *g_Ruminococcaceae UCG-010* | ＜0.001^c^ | 0.002^c^ | ＜0.001^c^ | 2.421^bc^ | 2.466^bc^ | 2.293^bc^ | 8.822^a^ | 7.996^a^ |  |  |
| *g_Faecalibacterium* | 0.039^b^ | 0.002^b^ | 23.728^a^ | 0.740^b^ | 0.180^b^ | 0.192^b^ | 0.017^b^ | 0.019^b^ |  |  |
| *g_unclassified Ruminococcaceae* | 1.583^abcd^ | 0.913^bcd^ | 1.718^abcd^ | 3.026^abc^ | 1.901^abcd^ | 4.091^a^ | 2.771^abcd^ | 4.382^a^ |  |  |
| *g_norank Muribaculaceae* | 0.007 | 0.002 | 0.002 | 0.296 | 3.583 | 3.311 | 0.574 | 0.442 |  |  |
| *g_[Eubacterium] coprostanoligenes* | 0.008^d^ | 0.592^d^ | 0.005^d^ | 4.034^ab^ | 1.750^bcd^ | 3.284^abc^ | 4.724^a^ | 5.230^a^ |  |  |
| *g_Lactobacillus* | 0.057^c^ | 10.036^b^ | 2.246^c^ | 0.215^c^ | 1.208^c^ | ＜0.001^c^ | ＜0.001^c^ | ＜0.001^c^ |  |  |
| *g_Clostridium_sensu_stricto_1* | 2.612 | 14.894 | 1.063 | 0.027 | 1.166 | 0.427 | 0.049 | 0.025 |  |  |
| *g_Ruminococcaceae NK4A214* | 0.002^d^ | 0.005^d^ | 0.012^d^ | 0.580^d^ | 1.162^d^ | 1.278^d^ | 1.162^d^ | 0.952^d^ |  |  |
| *g_Variovorax* | 18.797^a^ | 0.177^c^ | 0.170^c^ | 0.145^c^ | 0.242^c^ | ＜0.001^c^ | ＜0.001^c^ | ＜0.001^c^ |  |  |
| *g_Treponema_2* | ＜0.001^b^ | ＜0.001^b^ | 0.002^b^ | 0.002^b^ | 0.019^b^ | 2.125^b^ | 1.142^b^ | 0.594^b^ |  |  |
| *g_Stenotrophomonas* | 17.204^a^ | 0.232^c^ | 0.128^c^ | 0.091^c^ | 0.192^c^ | ＜0.001^c^ | 0.002^c^ | ＜0.001^c^ |  |  |
| *g_Ruminococcaceae UCG-014* | 0.015 | 0.214 | 0.987 | 0.728 | 1.292 | 3.060 | 1.775 | 0.900 |  |  |
| *g_Alistipes* | ＜0.001^d^ | ＜0.001^d^ | 0.003^d^ | 0.025^d^ | 0.161^d^ | 5.053^ab^ | 2.599^c^ | 5.711^a^ |  |  |
| *g_norank_Barnesiellaceae* | 0.003 | ＜0.001 | ＜0.001 | 6.514 | 2.202 | 1.129 | 1.280 | 0.733 |  |  |
| *g_Fournierella* | 0.513 | 1.795 | 7.851 | 0.187 | 0.195 | 0.918 | 0.007 | ＜0.001 |  |  |

**Table S6 Changes in the relative abundance of bacterial taxa in mucosa samples according to gastrointestinal tract region (GIT), age and interaction (GIT*age).**

|  | **Region** | | | **SEM** | ***P*** |
| --- | --- | --- | --- | --- | --- |
| **Taxa** | **Rumen** | **Cecum** | **colon** |  |  |
| p_Firmicutes | 28.237^b^ | 50.508^a^ | 49.000^a^ | 3.623 | ＜0.001 |
| p_Bacteroidetes | 37.094^a^ | 17.243^b^ | 20.621^b^ | 2.715 | ＜0.001 |
| p_Proteobacteria | 19.816^a^ | 9.995^b^ | 9.873^b^ | 2.923 | 0.046 |
| p_Spirochaetes | 1.417^b^ | 17.637^a^ | 14.302^a^ | 2.577 | ＜0.001 |
| p_Epsilonbacteraeota | 7.287^a^ | 0.540^b^ | 0.807^b^ | 1.722 | 0.012 |
| p_Patescibacteria | 1.101 | 0.838 | 1.281 | 0.365 | 0.700 |
| *g_Treponema_2* | 1.253^b^ | 17.483^a^ | 14.007^a^ | 2.584 | ＜0.001 |
| *g_Bacteroides* | 0.387^b^ | 9.096^a^ | 8.248^a^ | 1.999 | 0.008 |
| *g_Escherichia-Shigella* | 0.377 | 8.534 | 4.396 | 2.302 | 0.057 |
| *g_unclassified Lachnospiraceae* | 0.500^b^ | 6.762^a^ | 4.922^a^ | 1.277 | 0.006 |
| *g_Butyrivibrio_2* | 10.919^a^ | 0.038^b^ | 0.298^b^ | 1.932 | ＜0.001 |
| *g_Prevotella_1* | 9.008^a^ | 1.117^b^ | 0.982^b^ | 1.136 | ＜0.001 |
| *g_Prevotellaceae_UCG-001* | 10.660^a^ | 0.160^b^ | 0.099^b^ | 1.618 | ＜0.001 |
| *g_Ruminococcaceae_UCG-005* | 0.224^b^ | 4.726^a^ | 5.199^a^ | 0.574 | ＜0.001 |
| *g_Campylobacter* | 7.256^a^ | 0.517^b^ | 0.744^b^ | 1.715 | 0.011 |
| *g_Rikenellaceae_RC9_gut_group* | 3.139 | 2.139 | 2.896 | 0.519 | 0.385 |
| *g_norank Neisseriaceae* | 7.036^a^ | 0.012^b^ | 0.021^b^ | 1.340 | ＜0.001 |
| *g_Clostridium_sensu_stricto_1* | 0.030 | 4.008 | 2.606 | 1.564 | 0.208 |
| *g_Ruminococcaceae_UCG-010* | 0.190^b^ | 2.826^a^ | 3.431^a^ | 0.356 | ＜0.001 |
| *g_Christensenellaceae_R-7_group* | 1.527 | 2.168 | 2.272 | 0.308 | 0.193 |
| *g_Butyricicoccus* | 0.021^b^ | 2.518^a^ | 2.666^a^ | 0.523 | 0.001 |
| *g_norank Muribaculaceae* | 2.628 | 0.738 | 1.259 | 0.800 | 0.243 |
| *g_unclassified Pasteurellaceae* | 3.943^a^ | 0.009^b^ | 0.003^b^ | 1.276 | 0.048 |
| *g_Faecalibacterium* | 0.016^b^ | 2.056^a^ | 1.510^a^ | 0.935 | 0.006 |
| *g_Subdoligranulum* | 0.010^b^ | 2.220^a^ | 1.352^a^ | 1.173 | 0.022 |
| *g_unclassified Ruminococcaceae* | 0.240^b^ | 1.366^a^ | 1.773^a^ | 0.289 | 0.002 |
| *g_norank F082* | 2.430^a^ | 0.265^b^ | 0.294^b^ | 0.492 | 0.004 |

|  | **Age** | | | | | | | | **SEM** | ***P*** |
| --- | --- | --- | --- | --- | --- | --- | --- | --- | --- | --- |
| **Taxa** | **0d** | **14d** | **28d** | **42d** | **56d** | **4m** | **6m** | **18m** |  |  |
| p_Firmicutes | 15.439^c^ | 46.419^ab^ | 46.971^ab^ | 47.774^ab^ | 58.153^a^ | 41.842^ab^ | 31.691^bc^ | 48.467^ab^ | 5.916 | 0.001 |
| p_Bacteroidetes | 15.866^de^ | 4.535^e^ | 36.507^ab^ | 45.858^a^ | 30.157^bc^ | 23.469^bcd^ | 26.616^bcd^ | 16.728^cde^ | 4.434 | ＜0.001 |
| p_Proteobacteria | 46.578^a^ | 36.454^a^ | 9.879^b^ | 2.156^b^ | 4.412^b^ | 2.942^b^ | 3.473^b^ | 3.995^b^ | 4.773 | ＜0.001 |
| p_Spirochaetes | 1.310^b^ | 0.034^b^ | 0.345^b^ | 0.538^b^ | 0.396^b^ | 27.593^a^ | 34.760^a^ | 22.160^a^ | 4.208 | ＜0.001 |
| p_Epsilonbacteraeota | 0.258 | 9.784 | 4.398 | 1.188 | 2.253 | 1.027 | 0.615 | 3.472 | 2.812 | 0.306 |
| p_Patescibacteria | 6.330^a^ | 0.033^b^ | 0.013^b^ | 0.072^b^ | 0.186^b^ | 0.952^b^ | 0.375^b^ | 0.686^b^ | 0.597 | ＜0.001 |
| *g_Treponema_2* | 0.002^b^ | 0.015^b^ | 0.319^b^ | 0.535^b^ | 0.390^b^ | 27.516^a^ | 34.478^a^ | 22.177^a^ | 4.219 | ＜0.001 |
| *g_Bacteroides* | 0.035^c^ | 1.207^c^ | 22.395^a^ | 13.834^ab^ | 6.071^bc^ | 1.627^c^ | 0.660^c^ | 0.446^c^ | 3.264 | ＜0.001 |
| *g_Escherichia-Shigella* | 8.159^b^ | 19.538^a^ | 2.139^b^ | 0.376^b^ | 2.486^b^ | 0.259^b^ | 0.016^b^ | 0.002^b^ | 3.759 | 0.004 |
| *g_unclassified Lachnospiraceae* | 0.017^b^ | 2.392^b^ | 2.818^b^ | 6.663^b^ | 16.130^a^ | 2.247^b^ | 0.692^b^ | 0.602^b^ | 2.086 | ＜0.001 |
| *g_Butyrivibrio_2* | ＜0.001 | 1.938 | 1.223 | 1.226 | 6.833 | 7.809 | 2.626 | 8.355 | 3.155 | 0.380 |
| *g_Prevotella_1* | 0.015^c^ | 0.064^c^ | 1.264^c^ | 11.583^a^ | 4.237^bc^ | 1.679^c^ | 8.827^ab^ | 1.826^c^ | 1.856 | ＜0.001 |
| *g_Prevotellaceae_UCG-001* | 0.008 | 0.571 | 2.971 | 4.418 | 8.329 | 5.195 | 4.209 | 3.398 | 2.642 | 0.476 |
| *g_Ruminococcaceae_UCG-005* | 0.016^c^ | 0.047^c^ | 0.201^c^ | 9.388^a^ | 3.172^b^ | 4.469^b^ | 4.498^b^ | 4.748^b^ | 0.937 | ＜0.001 |
| *g_Campylobacter* | 0.004 | 9.709 | 4.388 | 1.188 | 2.252 | 1.027 | 0.615 | 3.472 | 2.800 | 0.300 |
| *g_Rikenellaceae_RC9_gut_group* | 0.020^c^ | 0.219^c^ | 0.887^c^ | 2.643^bc^ | 1.629^c^ | 5.323^a^ | 6.045^a^ | 5.033^ab^ | 0.847 | ＜0.001 |
| *g_norank Neisseriaceae* | 0.017^b^ | 12.699^a^ | 6.010^b^ | 0.073^b^ | 0.034^b^ | 0.002^b^ | 0.001^b^ | 0.016^b^ | 2.188 | 0.001 |
| *g_Clostridium_sensu_stricto_1* | 0.484^b^ | 13.674^a^ | 1.949^b^ | 0.058^b^ | 0.491^b^ | 0.749^b^ | 0.071^b^ | 0.113^b^ | 2.554 | 0.006 |
| *g_Ruminococcaceae_UCG-010* | 0.039^c^ | 0.079^c^ | 0.149^c^ | 2.970^b^ | 2.512^b^ | 1.991^b^ | 5.385^a^ | 3.758^ab^ | 0.581 | ＜0.001 |
| *g_Christensenellaceae_R-7_group* | 0.149^d^ | 0.422^cd^ | 0.678^cd^ | 1.963^bc^ | 1.236^bcd^ | 4.096^a^ | 2.260^b^ | 5.117^a^ | 0.503 | ＜0.001 |
| *g_Butyricicoccus* | 0.840^b^ | 7.330^a^ | 5.209^a^ | 0.280^b^ | 0.025^b^ | 0.009^b^ | ＜0.001^b^ | ＜0.001^b^ | 0.854 | ＜0.001 |
| *g_norank Muribaculaceae* | 0.011^b^ | 0.019^b^ | 0.038^b^ | 3.011^ab^ | 6.043^a^ | 1.695^b^ | 0.516^b^ | 1.003^b^ | 1.306 | 0.026 |
| *g_unclassified Pasteurellaceae* | 10.436 | 0.104 | 0.008 | 0.007 | 0.001 | ＜0.001 | ＜0.001 | ＜0.001 | 3.717 | 0.547 |
| *g_Faecalibacterium* | 0.006^b^ | 0.053^b^ | 9.158^a^ | 0.170^b^ | 0.105^b^ | 0.052^b^ | 0.003^b^ | 0.001^b^ | 1.526 | 0.001 |
| *g_Subdoligranulum* | 0.010^b^ | 7.075^a^ | 2.431^ab^ | 0.020^b^ | 0.009^b^ | 0.001^b^ | 0.002^b^ | 0.001^b^ | 1.915 | 0.026 |
| *g_unclassified Ruminococcaceae* | 0.496 | 1.050 | 1.502 | 1.831 | 0.891 | 1.062 | 0.860 | 1.244 | 0.472 | 0.653 |
| *g_norank F082* | 0.044^b^ | 0.515^b^ | 4.225^a^ | 0.312^b^ | 0.371^b^ | 0.661^b^ | 0.588^b^ | 1.231^b^ | 0.804 | 0.016 |
|  | **Region*Age** | | | | | | | | **SEM** | ***P*** |
| **Taxa** | **Rumen_0d** | **Rumen_14d** | **Rumen_28d** | **Rumen_42d** | **Rumen_56d** | **Rumen_4m** | **Rumen_ 6m** | **Rumen_ 18m** |  |  |
| p_Firmicutes | 10.043^g^ | 10.874^fg^ | 16.425^efg^ | 21.773^defg^ | 40.017^abcdefg^ | 47.107^abcde^ | 25.439^cdefg^ | 54.220^abcd^ | 10.247 | ＜0.001 |
| p_Bacteroidetes | 10.924^fgh^ | 9.277^gh^ | 42.481^abcd^ | 66.323^a^ | 48.578^abc^ | 38.287^bcde^ | 55.039^ab^ | 25.841^cdefgh^ | 7.679 | ＜0.001 |
| p_Proteobacteria | 56.580^a^ | 49.345^ab^ | 22.814^bcde^ | 4.170^cde^ | 2.974^cde^ | 6.374^cde^ | 9.183^cde^ | 7.087^cde^ | 8.267 | ＜0.001 |
| p_Spirochaetes | 0.703^c^ | 0.059^c^ | 0.974^c^ | 1.191^c^ | 0.764^c^ | 0.370^c^ | 6.081^c^ | 1.198^c^ | 7.288 | 0.004 |
| p_Epsilonbacteraeota | 0.116 | 29.094 | 13.007 | 3.529 | 3.793 | 2.814 | 1.642 | 4.300 | 4.871 | 0.074 |
| p_Patescibacteria | 3.444^bc^ | 0.003^c^ | 0.040^c^ | 0.182^c^ | 0.153^c^ | 2.562^c^ | 0.776^c^ | 1.645^c^ | 1.033 | ＜0.001 |
| *g_Treponema_2* | 0.005^c^ | 0.034^c^ | 0.900^c^ | 1.183^c^ | 0.749^c^ | 0.173^c^ | 5.844^c^ | 1.134^c^ | 7.308 | ＜0.001 |
| *g_Bacteroides* | 0.067^c^ | 1.961^c^ | 0.892^c^ | 0.049^c^ | 0.123^c^ | 0.002^c^ | ＜0.001^c^ | 0.002^c^ | 5.653 | ＜0.001 |
| *g_Escherichia-Shigella* | 1.215^b^ | 1.628^b^ | 0.020^b^ | 0.091^b^ | 0.062^b^ | ＜0.001^b^ | ＜0.001^b^ | ＜0.001^b^ | 6.510 | 0.024 |
| *g_unclassified Lachnospiraceae* | 0.030^c^ | 0.042^c^ | 0.204^c^ | 0.266^c^ | 1.898^c^ | 0.426^c^ | 0.303^c^ | 0.833^c^ | 3.613 | ＜0.001 |
| *g_Butyrivibrio_2* | ＜0.001^d^ | 5.809^bcd^ | 3.662^cd^ | 3.474^cd^ | 20.382^abc^ | 23.405^a^ | 7.831^abcd^ | 22.789^ab^ | 5.465 | 0.038 |
| *g_Prevotella_1* | 0.039^b^ | 0.148^b^ | 3.745^b^ | 26.524^a^ | 7.210^b^ | 4.579^b^ | 25.923^a^ | 3.894^b^ | 3.214 | ＜0.001 |
| *g_Prevotellaceae_UCG-001* | 0.017^b^ | 1.672^b^ | 8.907^b^ | 12.003^ab^ | 24.796^a^ | 15.530^ab^ | 12.499^ab^ | 9.853^b^ | 4.576 | 0.015 |
| *g_Ruminococcaceae_UCG-005* | 0.035^e^ | 0.121^e^ | 0.528^e^ | 0.153^e^ | 0.236^e^ | 0.350^e^ | 0.096^e^ | 0.035^e^ | 1.623 | ＜0.001 |
| *g_Campylobacter* | 0.007 | 28.980 | 12.980 | 3.529 | 3.793 | 2.814 | 1.642 | 4.300 | 4.850 | 0.071 |
| *g_Rikenellaceae_RC9_gut_group* | 0.052^f^ | 0.592^f^ | 2.631^bcdef^ | 6.249^abc^ | 1.408^cdef^ | 5.990^abcd^ | 3.501^bcdef^ | 4.687^abcdef^ | 1.467 | ＜0.001 |
| *g_norank Neisseriaceae* | 0.044^c^ | 37.944^a^ | 17.933^b^ | 0.197^c^ | 0.059^c^ | 0.002^c^ | 0.002^c^ | 0.049^c^ | 3.790 | ＜0.001 |
| *g_Clostridium_sensu_stricto_1* | 0.109^b^ | 0.050^b^ | 0.010^b^ | 0.035^b^ | 0.035^b^ | ＜0.001^b^ | ＜0.001^b^ | ＜0.001^b^ | 4.424 | 0.054 |
| *g_Ruminococcaceae_UCG-010* | 0.042^e^ | 0.140^e^ | 0.439^de^ | 0.024^e^ | 0.084^e^ | 0.207^e^ | 0.167^e^ | 0.417^de^ | 1.007 | ＜0.001 |
| *g_Christensenellaceae_R-7_group* | 0.138^e^ | 1.146^de^ | 1.982^bcde^ | 0.880^de^ | 0.738^de^ | 4.463^abc^ | 0.939^de^ | 1.931^bcde^ | 0.872 | ＜0.001 |
| *g_Butyricicoccus* | 0.017^d^ | 0.030^d^ | 0.015^d^ | 0.061^d^ | 0.047^d^ | ＜0.001^d^ | ＜0.001^d^ | ＜0.001^d^ | 1.478 | ＜0.001 |
| *g_norank Muribaculaceae* | 0.007 | 0.012 | 0.111 | 6.204 | 10.426 | 2.333 | 0.506 | 1.427 | 2.263 | 0.305 |
| *g_unclassified Pasteurellaceae* | 31.212 | 0.296 | 0.024 | 0.013 | 0.002 | ＜0.001 | ＜0.001 | ＜0.001 | 6.439 | 0.509 |
| *g_Faecalibacterium* | 0.010^b^ | 0.010^b^ | 0.040^b^ | 0.010^b^ | 0.056^b^ | ＜0.001^b^ | ＜0.001^b^ | ＜0.001^b^ | 2.643 | 0.011 |
| *g_Subdoligranulum* | 0.007 | ＜0.001 | 0.008 | 0.042 | 0.022 | ＜0.001 | ＜0.001 | ＜0.001 | 3.317 | 0.503 |
| *g_unclassified Ruminococcaceae* | 0.227 | 0.350 | 0.293 | 0.037 | 0.416 | 0.209 | 0.106 | 0.283 | 0.817 | 0.362 |
| *g_norank F082* | 0.116^b^ | 1.028^b^ | 12.411^a^ | 0.848^b^ | 0.762^b^ | 1.753^b^ | 1.095^b^ | 1.423^b^ | 1.393 | ＜0.001 |
|  | **Cecum_0d** | **Cecum_14d** | **Cecum_28d** | **Cecum_42d** | **Cecum_56d** | **Cecum_4m** | **Cecum_6m** | **Cecum_18m** |  |  |
| p_Firmicutes | 25.630^cdefg^ | 64.071^a^ | 60.908^ab^ | 60.098^abc^ | 69.375^a^ | 42.352^abcdefg^ | 27.281^bcdefg^ | 46.058^abcdef^ |  |  |
| p_Bacteroidetes | 11.572^fgh^ | 1.191^h^ | 35.429^bcdefg^ | 36.642^bcdef^ | 16.271^efgh^ | 10.753^fgh^ | 9.950^gh^ | 14.243^efgh^ |  |  |
| p_Proteobacteria | 48.062^ab^ | 29.483^bcd^ | 3.137^cde^ | 1.285^de^ | 6.894^cde^ | 1.484^de^ | 0.673^de^ | 1.632^de^ |  |  |
| p_Spirochaetes | 1.055^c^ | 0.007^c^ | 0.008^c^ | 0.326^c^ | 0.178^c^ | 42.165^ab^ | 59.497^a^ | 32.329^b^ |  |  |
| p_Epsilonbacteraeota | 0.192 | 0.121 | 0.135 | 0.024 | 1.728 | 0.188 | 0.086 | 1.731 |  |  |
| p_Patescibacteria | 5.766^b^ | 0.091^c^ | ＜0.001^c^ | 0.017^c^ | 0.207^c^ | 0.249^c^ | 0.167^c^ | 0.207^c^ |  |  |
| *g_Treponema_2* | ＜0.001^c^ | 0.005^c^ | 0.008^c^ | 0.326^c^ | 0.175^c^ | 42.155^ab^ | 59.066^a^ | 32.299^b^ |  |  |
| *g_Bacteroides* | 0.035^c^ | 0.626^c^ | 35.345^a^ | 22.296^ab^ | 8.019^bc^ | 1.953^c^ | 0.594^c^ | 0.876^c^ |  |  |
| *g_Escherichia-Shigella* | 30.767^a^ | 27.600^a^ | 2.978^b^ | 0.612^b^ | 5.672^b^ | 0.639^b^ | 0.005^b^ | 0.002^b^ |  |  |
| *g_unclassified Lachnospiraceae* | 0.020^c^ | 2.300^c^ | 6.313^bc^ | 9.918^bc^ | 30.157^a^ | 2.056^c^ | 0.476^c^ | 0.606^c^ |  |  |
| *g_Butyrivibrio_2* | ＜0.001^d^ | 0.002^d^ | 0.002^d^ | 0.040^d^ | 0.071^d^ | 0.010^d^ | 0.039^d^ | 0.130^d^ |  |  |
| *g_Prevotella_1* | 0.003^b^ | 0.020^b^ | 0.008^b^ | 4.811^b^ | 1.933^b^ | 0.146^b^ | 0.353^b^ | 1.289^b^ |  |  |
| *g_Prevotellaceae_UCG-001* | 0.003^b^ | 0.010^b^ | 0.002^b^ | 0.749^b^ | 0.106^b^ | 0.030^b^ | 0.114^b^ | 0.217^b^ |  |  |
| *g_Ruminococcaceae_UCG-005* | 0.010^e^ | 0.008^e^ | 0.029^e^ | 16.267^a^ | 2.974^de^ | 5.674^cd^ | 4.386^cde^ | 6.887^bcd^ |  |  |
| *g_Campylobacter* | 0.003 | 0.074 | 0.135 | 0.024 | 1.726 | 0.188 | 0.086 | 1.731 |  |  |
| *g_Rikenellaceae_RC9_gut_group* | ＜0.001^f^ | 0.007^f^ | 0.008^f^ | 0.814^ef^ | 1.046^def^ | 3.438^bcdef^ | 5.627^abcde^ | 6.169^abc^ |  |  |
| *g_norank Neisseriaceae* | 0.003^c^ | 0.042^c^ | 0.013^c^ | 0.012^c^ | 0.022^c^ | ＜0.001^c^ | ＜0.001^c^ | ＜0.001^c^ |  |  |
| *g_Clostridium_sensu_stricto_1* | 1.638^b^ | 24.843^a^ | 3.289^b^ | 0.067^b^ | 0.755^b^ | 1.236^b^ | 0.077^b^ | 0.158^b^ |  |  |
| *g_Ruminococcaceae_UCG-010* | 0.030^e^ | 0.010^e^ | 0.003^e^ | 4.327^bc^ | 2.187^cde^ | 3.575^bcd^ | 6.223^b^ | 5.324^bc^ |  |  |
| *g_Christensenellaceae_R-7_group* | 0.081^e^ | 0.029^e^ | 0.010^e^ | 2.217^bcde^ | 1.420^de^ | 4.624^ab^ | 2.321^bcde^ | 6.643^a^ |  |  |
| *g_Butyricicoccus* | 3.336^cd^ | 9.252^ab^ | 7.552^bc^ | 0.234^d^ | 0.025^d^ | 0.017^d^ | ＜0.001^d^ | ＜0.001^d^ |  |  |
| *g_norank Muribaculaceae* | 0.008 | 0.015 | ＜0.001 | 1.045 | 2.796 | 0.871 | 0.402 | 0.767 |  |  |
| *g_unclassified Pasteurellaceae* | 0.086 | 0.002 | ＜0.001 | 0.005 | 0.002 | ＜0.001 | ＜0.001 | ＜0.001 |  |  |
| *g_Faecalibacterium* | 0.008^b^ | 0.012^b^ | 15.973^a^ | 0.175^b^ | 0.160^b^ | 0.113^b^ | 0.003^b^ | 0.002^b^ |  |  |
| *g_Subdoligranulum* | 0.030 | 14.558 | 3.158 | 0.003 | 0.005 | 0.002 | 0.002 | 0.003 |  |  |
| *g_unclassified Ruminococcaceae* | 0.707 | 0.658 | 2.056 | 2.182 | 0.769 | 1.696 | 1.033 | 1.607 |  |  |
| *g_norank F082* | ＜0.001^b^ | 0.109^b^ | 0.017^b^ | 0.049^b^ | 0.158^b^ | 0.086^b^ | 0.276^b^ | 1.339^b^ |  |  |
|  | **Colon_0d** | **Colon_14d** | **Colon_28d** | **Colon_42d** | **Colon_56d** | **Colon_4m** | **Colon_6m** | **Colon_18m** |  |  |
| p_Firmicutes | 14.042^efg^ | 64.313^a^ | 63.578^a^ | 61.452^ab^ | 65.067^a^ | 36.067^abcdefg^ | 42.355^abcdefg^ | 45.124^abcdefg^ |  |  |
| p_Bacteroidetes | 23.669^cdefgh^ | 3.137^h^ | 31.609^bcdefg^ | 34.608^bcdefg^ | 25.622^cdefgh^ | 21.366^defgh^ | 14.859^efgh^ | 10.100^fgh^ |  |  |
| p_Proteobacteria | 35.588^ab^ | 30.536^abc^ | 3.686^cde^ | 1.013^de^ | 3.368^cde^ | 0.969^de^ | 0.564^e^ | 3.265^cde^ |  |  |
| p_Spirochaetes | 2.086^c^ | 0.037^c^ | 0.052^c^ | 0.096^c^ | 0.246^c^ | 40.244^ab^ | 38.703^ab^ | 32.953^b^ |  |  |
| p_Epsilonbacteraeota | 0.444 | 0.136 | 0.052 | 0.010 | 1.236 | 0.077 | 0.116 | 4.386 |  |  |
| p_Patescibacteria | 9.592^a^ | 0.003^c^ | ＜0.001^c^ | 0.019^c^ | 0.199^c^ | 0.044^c^ | 0.183^c^ | 0.207^c^ |  |  |
| *g_Treponema_2* | ＜0.001^c^ | 0.007^c^ | 0.049^c^ | 0.096^c^ | 0.246^c^ | 40.220^ab^ | 38.523^ab^ | 32.920^b^ |  |  |
| *g_Bacteroides* | 0.003^c^ | 1.035^c^ | 30.948^a^ | 19.156^abc^ | 10.070^bc^ | 2.925^c^ | 1.386^c^ | 0.459^c^ |  |  |
| *g_Escherichia-Shigella* | 0.030^b^ | 29.385^a^ | 3.418^b^ | 0.424^b^ | 1.724^b^ | 0.136^b^ | 0.044^b^ | 0.005^b^ |  |  |
| *g_unclassified Lachnospiraceae* | 0.002^c^ | 4.833^c^ | 1.938^c^ | 9.806^bc^ | 16.874^b^ | 4.259^c^ | 1.297^c^ | 0.367^c^ |  |  |
| *g_Butyrivibrio_2* | ＜0.001^d^ | 0.005^d^ | 0.005^d^ | 0.165^d^ | 0.045^d^ | 0.012^d^ | 0.008^d^ | 2.145^d^ |  |  |
| *g_Prevotella_1* | ＜0.001^b^ | 0.024^b^ | 0.040^b^ | 3.415^b^ | 3.568^b^ | 0.311^b^ | 0.205^b^ | 0.294^b^ |  |  |
| *g_Prevotellaceae_UCG-001* | 0.003^b^ | 0.030^b^ | 0.005^b^ | 0.503^b^ | 0.084^b^ | 0.025^b^ | 0.015^b^ | 0.124^b^ |  |  |
| *g_Ruminococcaceae_UCG-005* | ＜0.001^e^ | 0.010^e^ | 0.047^e^ | 11.744^ab^ | 6.307^cd^ | 7.383^bcd^ | 9.013^bc^ | 7.087^bcd^ |  |  |
| *g_Campylobacter* | 0.003 | 0.072 | 0.050 | 0.010 | 1.236 | 0.077 | 0.116 | 4.386 |  |  |
| *g_Rikenellaceae_RC9_gut_group* | ＜0.001^f^ | 0.059^f^ | 0.022^f^ | 0.865^ef^ | 2.433^bcdef^ | 6.539^ab^ | 9.007^a^ | 4.243^abcdef^ |  |  |
| *g_norank Neisseriaceae* | ＜0.001^c^ | 0.111^c^ | 0.024^c^ | 0.010^c^ | 0.020^c^ | 0.003^c^ | ＜0.001^c^ | ＜0.001^c^ |  |  |
| *g_Clostridium_sensu_stricto_1* | 0.091^b^ | 16.128^a^ | 2.547^b^ | 0.072^b^ | 0.683^b^ | 1.009^b^ | 0.135b | 0.180^b^ |  |  |
| *g_Ruminococcaceae_UCG-010* | 0.042^e^ | 0.086^e^ | 0.005^e^ | 4.561^bc^ | 5.265^bc^ | 2.190^cde^ | 9.765^a^ | 5.531^bc^ |  |  |
| *g_Christensenellaceae_R-7_group* | 0.205^e^ | 0.093^e^ | 0.042^e^ | 2.791^bcde^ | 1.551^cde^ | 3.201^cd^ | 3.519^bcd^ | 6.776^a^ |  |  |
| *g_Butyricicoccus* | ＜0.001^d^ | 12.708^a^ | 8.060^b^ | 0.547^d^ | 0.003^d^ | 0.010^d^ | ＜0.001^d^ | ＜0.001^d^ |  |  |
| *g_norank Muribaculaceae* | 0.017 | 0.029 | 0.003 | 1.785 | 4.905 | 1.881 | 0.641 | 0.814 |  |  |
| *g_unclassified Pasteurellaceae* | 0.010 | 0.013 | ＜0.001 | 0.002 | ＜0.001 | ＜0.001 | ＜0.001 | ＜0.001 |  |  |
| *g_Faecalibacterium* | 0.002^b^ | 0.138^b^ | 11.461^a^ | 0.325^b^ | 0.099^b^ | 0.044^b^ | 0.007^b^ | 0.002^b^ |  |  |
| *g_Subdoligranulum* | ＜0.001 | 6.667 | 4.127 | 0.015 | ＜0.001 | ＜0.001 | 0.005 | ＜0.001 |  |  |
| *g_unclassified Ruminococcaceae* | 0.555 | 2.141 | 2.158 | 3.274 | 1.489 | 1.282 | 1.442 | 1.842 |  |  |
| *g_norank F082* | ＜0.001^b^ | 0.407^b^ | 0.247^b^ | 0.039^b^ | 0.192^b^ | 0.145^b^ | 0.394^b^ | 0.930^b^ |  |  |

Table S7 The 13 predominant KEGG othologs pathways(kegg level3) in digesta and mucosa sample of rumen, cecum and colon at the different ages (the average abundance≥1.5%). The data showed in sheet1.

Table S8 Comparison of predominant bacterial function (kegg level3) among rumen, cecum and colon (average proportion≥1.5%).

| **Items** | **Rumen** | **Cecum** | **Colon** | **SEM** | ***P*** |
| --- | --- | --- | --- | --- | --- |
| **Digesta** |  |  |  |  |  |
| ko01230 | 5.996 | 6.233 | 6.243 | 0.073 | 0.297 |
| ko01200 | 4.133 | 4.079 | 4.068 | 0.013 | 0.072 |
| ko02010 | 2.790^b^ | 3.896^a^ | 3.8335^a^ | 0.107 | ＜0.001 |
| ko03010 | 3.636 | 3.344 | 3.323 | 0.063 | 0.073 |
| ko00230 | 3.215^a^ | 3.000^b^ | 2.973^b^ | 0.030 | 0.001 |
| ko00240 | 2.823 | 2.651 | 2.638 | 0.037 | 0.076 |
| ko02024 | 1.939^b^ | 2.264^a^ | 2.262^a^ | 0.040 | ＜0.001 |
| ko02020 | 1.854^b^ | 2.231^a^ | 2.267^a^ | 0.064 | 0.012 |
| ko00520 | 1.737 | 1.726 | 1.714 | 0.032 | 0.960 |
| ko00010 | 1.716 | 1.668 | 1.644 | 0.014 | 0.111 |
| ko00620 | 1.532^b^ | 1.645^a^ | 1.648^a^ | 0.012 | ＜0.001 |
| ko00970 | 1.640 | 1.565 | 1.554 | 0.024 | 0.269 |
| Ko00190 | 1.626^a^ | 1.501^b^ | 1.495^b^ | 0.019 | 0.005 |
| **Mucosa** |  |  |  |  |  |
| ko01230 | 6.100 | 6.211 | 6.202 | 0.057 | 0.683 |
| ko01200 | 4.145^a^ | 3.951^b^ | 4.017^b^ | 0.019 | ＜0.001 |
| ko02010 | 3.145^b^ | 4.223^a^ | 3.919^a^ | 0.115 | ＜0.001 |
| ko03010 | 3.596 | 3.387 | 3.399 | 0.050 | 0.156 |
| ko00230 | 3.195^a^ | 3.077^b^ | 3.056^b^ | 0.020 | 0.007 |
| ko00240 | 2.679 | 2.703 | 2.667 | 0.027 | 0.862 |
| ko02024 | 1.943^b^ | 2.244^a^ | 2.219^a^ | 0.034 | ＜0.001 |
| ko02020 | 2.036^b^ | 2.336^a^ | 2.321^a^ | 0.056 | 0.047 |
| ko00520 | 1.653 | 1.782 | 1.731 | 0.032 | 0.245 |
| ko00010 | 1.577^b^ | 1.673^a^ | 1.653^ab^ | 0.017 | 0.049 |
| ko00620 | 1.502^b^ | 1.615^a^ | 1.621^a^ | 0.011 | ＜0.001 |
| ko00970 | 1.610 | 1.567 | 1.573 | 0.019 | 0.628 |
| ko00190 | 1.766^a^ | 1.461^b^ | 1.529^b^ | 0.031 | ＜0.001 |

Note: (1) ko01230, Biosynthesis of amino acids; ko01200, Carbon metabolism; ko02010, ABC transporters; ko03010, Ribosome; ko00230, Purine metabolism; ko00240, Pyrimidine metabolism; ko02024, Quorum sensing; ko02020, Two-component system; ko00520, Amino sugar and nucleotide sugar metabolism; ko00010, Glycolysis / Gluconeogenesis; ko00620, Pyruvate metabolism; ko00970, Aminoacyl-tRNA biosynthesis; ko00190, Oxidative phosphorylation. (2) Mean values with different superscripted lowercase letters within the same row differ significantly (*P* < 0.05).

Table S9 Signiﬁcant difference of 13 predominant KEGG gene pathways of bacterial microbiota at different ages in digesta and mucosa of rumen, cecum, and colon. The data showed in sheet 2. Mean values with different superscripted lowercase letters within the same row differ signiﬁcantly (*P* < 0.05).

Table S10 Comparison of 13 predominant KEGG gene pathways (KEGG level 3) of bacterial microbiota between in digesta and mucosa sample of rumen, cecum, and colon. The data showed in sheet 3.
